# Discovery of abnormal lithium-storage sites in molybdenum dioxide electrodes

**DOI:** 10.1038/ncomms11049

**Published:** 2016-03-22

**Authors:** Jeong Kuk Shon, Hyo Sug Lee, Gwi Ok Park, Jeongbae Yoon, Eunjun Park, Gyeong Su Park, Soo Sung Kong, Mingshi Jin, Jae-Man Choi, Hyuk Chang, Seokgwang Doo, Ji Man Kim, Won-Sub Yoon, Chanho Pak, Hansu Kim, Galen D. Stucky

**Affiliations:** 1 Samsung Advanced Institute of Technology, Samsung Electronics Co., Ltd., Suwon 443-803, Republic of Korea; 2 Department of Chemistry, Sungkyunkwan University, Suwon 440-746, Republic of Korea; 3 Department of Energy Science, Sungkyunkwan University, Suwon 440-746, Republic of Korea; 4 Department of Energy Engineering, Hanyang University, Seoul 133-791, Republic of Korea; 5 Key Laboratory of Natural Resource of the Changbai Mountain and Functional Molecular (Yanbian University), Ministry of Education, Department of Chemistry, Park Road 977, Yanji City, Jilin Province 133002, China; 6 Fuel Cell Group, Corporate R&D Center, Samsung SDI Co., Ltd., Yongin 446-577, Republic of Korea; 7 Department of Chemistry and Biochemistry, University of California, Santa Barbara, California 93106, USA

## Abstract

Developing electrode materials with high-energy densities is important for the development of lithium-ion batteries. Here, we demonstrate a mesoporous molybdenum dioxide material with abnormal lithium-storage sites, which exhibits a discharge capacity of 1,814 mAh g^−1^ for the first cycle, more than twice its theoretical value, and maintains its initial capacity after 50 cycles. Contrary to previous reports, we find that a mechanism for the high and reversible lithium-storage capacity of the mesoporous molybdenum dioxide electrode is not based on a conversion reaction. Insight into the electrochemical results, obtained by *in situ* X-ray absorption, scanning transmission electron microscopy analysis combined with electron energy loss spectroscopy and computational modelling indicates that the nanoscale pore engineering of this transition metal oxide enables an unexpected electrochemical mass storage reaction mechanism, and may provide a strategy for the design of cation storage materials for battery systems.

## Abstract (web summary)

Electrode materials with high energy density are important for the development of Li-ion batteries. Here, the authors report a molybdenum dioxide anode with abnormal lithium storage sites, exhibiting a discharge capacity twice its theoretical value by utilizing two different storage mechanisms.

## Graphical abstract

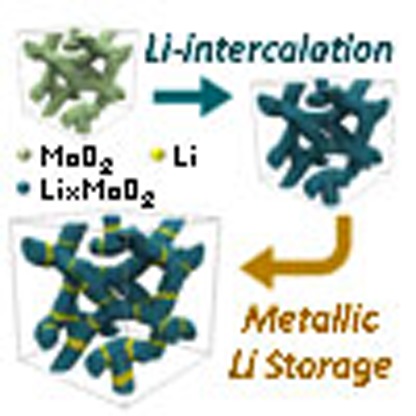

Lithium (Li)-ion batteries (LIBs) are a key-enabling technology for addressing the power and energy demands of electric vehicles and stationary electrical storage for renewable energy as well as mobile electronics^1^. However, the energy density of currently commercialized LIBs is already close to its technological limit^2^. In order to achieve the battery performance that all applications expect, much effort has been made to develop new electrode materials to improve both the energy density and cycle performance of LIBs^3^. The main goal of these research efforts are to enable energy densities that are higher than the theoretical limit predicted for current Li-ion intercalation batteries. These new batteries would have the desired physicochemical properties, especially high reversible capacity, structural flexibility and stability, high rate capability, low cost and environmental benignity^4^,^5^. In order to improve the performance of anode parts replacing graphite (theoretical capacity of 372 mAh g^−1^), there has been extensive research on developing anode materials such as transition metal oxides, silicon- or tin-based metal alloys, and related composite configurations^3^,^4^,^5^,^6^,^7^,^8^,^9^,^10^,^11^. Since they follow Li-storage mechanisms such as conversion and alloying reactions with Li, which are different from the Li intercalation reaction in the graphite anode, these newly developed anode materials show higher capacity than the current anode systems. In particular, there had been several reports that some transition metal oxides showed the reversible capacity exceeding their theoretical capacity based on the conversion reaction. This abnormal capacity of transition metal oxides has been explained by various mechanisms based on the interfacial reaction: (i) reversible formation/dissolution of organic film at the interface between the electrolyte and the transition metal oxide^9^,^12^; (ii) interfacial charge storage between metal nanocrystals and Li salts^13^,^14^; and (iii) generation of LiOH on the surface of metal oxide and subsequent reversible reaction between LiOH and Li^15^. Hence, even though the origins of extra capacity of transition metal oxides are different from each other, one of the effective approaches is nano-engineering of transition metal oxides for LIBs^16^,^17^,^18^. This approach has the potential to significantly increase the reversible capacity and the rate capability of anode materials by enlarging the interfacial area between the electrode and electrolyte. It is also expected that nanostructured transition metal oxides should show much improved cycle performance by providing the mechanical/structural integrity against huge changes in volume and crystal structure.

Mesoporous materials are excellent nanoscale-engineered candidates for numerous applications because of their high surface areas, tunable pore sizes, adjustable framework thickness and compositions, and diverse surface properties^19^,^20^,^21^,^22^,^23^. Recently, various novel mesoporous metal oxides have been widely investigated as electrode materials for LIBs^24^,^25^,^26^,^27^,^28^. These studies have opened up a possibility for the development of anode materials with significantly improved Li-storage performance. The mesostructure can be readily designed to exhibit hierarchical mesoporosity to promote facile and fast Li-ion diffusion, and a uniform framework thickness of 10 nm or less so that there is a reduced diffusion length for solid-state Li transport. Interestingly, we have found that the mesoporous MoO_2_ anode presented here gives a high Li-storage capacity (1,814 mAh g^−1^ at first cycle and 1,607 mAh g^−1^ after 50 cycles), which is much higher than its theoretical capacity based on the conversion reaction of MoO_2_ with Li (838 mAh g^−1^). As aforementioned, several research groups have newly proposed Li-storage mechanisms^12^,^13^,^14^,^15^,^29^; however, this extra Li-storage mechanism of transition metal oxide is still unclear, suggesting that the high capacity of the mesoporous MoO_2_ in the present work probably results from a different Li-storage mechanism than those previously reported^12^,^13^,^14^,^15^.

Here we report a mechanism for the high and reversible Li-storage capacity of the mesoporous MoO_2_ anode based on various physicochemical analyses and computational modelling. To the best of our knowledge, this is the first demonstration of a Li-storage oxide anode material that utilizes two different Li-storage mechanisms composed of both a Li-ion intercalation and a metallic Li storage. This enables a structural and electronic configuration that is, in a sense, a hybrid between the Li intercalation compound anode and the metallic Li anode, with the inherent safety features of the former and energy density approaching that of the latter. We believe that understanding the origin and mechanism of this excellent performance will enable important advances in the design and creation of high-energy storage devices.

## Results

### Li-storage characteristics of mesoporous MoO_2_

**Figure 1:**
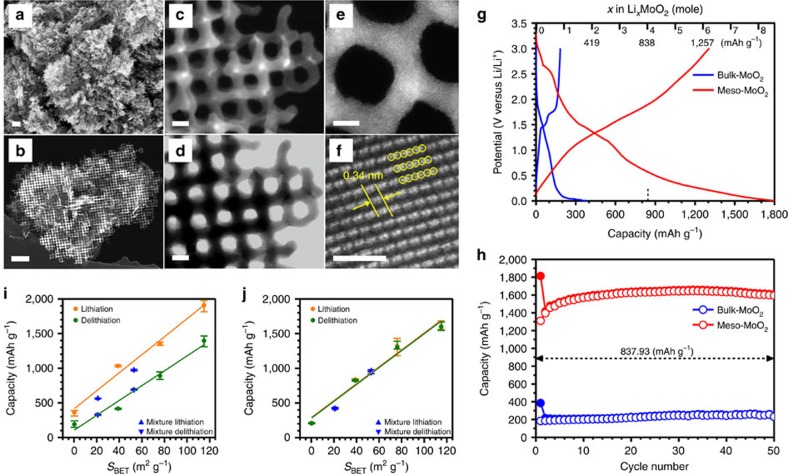
Ordered mesoporous MoO_2_ and Li-storage performance. (**a**,**b**) SEM, (**c**,**e**) STEM, (**d**) TEM and (**f**) HRTEM images of ordered mesoporous MoO_2_ materials. Electrochemical performance of ordered mesoporous MoO_2_ (*S*_BET_=115 m^2^ g^−1^) and bulk MoO_2_ (Aldrich, *S*_BET_=0.23 m^2^ g^−1^): (**g**) voltage profiles and (**h**) cycle performances at current rate of 0.1 C in 1.3 M LiPF_6_ (ethylene carbonate/diethyl carbonate (EC/DEC)=3/7, by volume ratio). The value (837.93 mAh g^−1^) represents the theoretical capacity for MoO_2_, based on conversion reaction (4 mol of Li per 1 mol of MoO_2_). Relationship of the Li-storage capacities of the MoO_2_ electrodes with respective to their surface areas: (**i**) the first and (**j**) the tenth lithiation/delithiation. The galvanostatic lithiation/delithiation tests were replicated for three times. Error bars (**i**,**j**) represent the standard deviation of the mean about a series of the measured values. Straight line is a linear fit. Scale bars= 100 nm (**a**,**b**), 10 nm (**c**,**d**), 5 nm (**e**) and 1.5 nm (**f**).

The mesoporous MoO_2_ material, which exhibits regular mesopores (18.2 nm in diameter), highly crystalline frameworks (∼7 nm thickness) and high surface areas of 115 m^2^ g^−1^, was successfully obtained from a mesoporous silica template (Fig. 1a–f and Supplementary Fig. 1). Electrochemical performances of the mesoporous MoO_2_ electrode and a bulk MoO_2_ (Aldrich, *S*_BET_=0.23 m^2^ g^−1^) electrode are shown in Fig. 1g,h. There is an obvious difference in the Li-storage behaviours of the mesoporous MoO_2_ and bulk MoO_2_ electrodes. For the bulk MoO_2_ electrode, the discharge (Li insertion) and charge (Li removal) capacities are 385 and 183 mAh g^−1^ (for the first cycle), respectively, indicating that 1.84 mol of Li per mol of MoO_2_ can be stored into bulk MoO_2_, and 0.87 mol of Li are reversibly released from the lithiated bulk MoO_2_. On the other hand, the mesoporous MoO_2_ delivered a reversible charge capacity of 1,308 mAh g^−1^ (for the first cycle) with an initial coulombic efficiency of 72.1% (discharge capacity of 1,814 mAh g^−1^), which is much higher than those of the bulk MoO_2_ electrode and corresponds to the reversible removal of 6.24 mol of Li per mol of MoO_2_ from the fully lithiated mesoporous MoO_2_. More importantly, the reversible Li-storage capacity of mesoporous MoO_2_ (6.24 mol of Li per mol of MoO_2_) at the first cycle exceeds the theoretical limit of Li-storage capacity through conversion reaction of MoO_2_ with Li (4 mol of Li per mol of MoO_2_), suggesting that a new Li-storage mechanism should be introduced to explain this unexpected Li-storage performance.

As shown in Fig. 1i,j, Supplementary Figs 2 and 3 and Supplementary Table 1, the Li-storage capacities of the MoO_2_ electrodes are linearly correlated with their surface areas, even though the shapes in the small-angle X-ray diffraction (XRD) patterns and N_2_ sorption isotherms of mesoporous MoO_2_ materials areas are very similar (Supplementary Figs 4 and 5). In the case of mesoporous MoO_2_ with surface area of 39 m^2^ g^−1^, the first and tenth reversible charge capacities are only 422 mAh g^−1^ (discharge capacity of 1,022 mAh g^−1^) and 814 mAh g^−1^, respectively, which are much lower than those of the mesoporous MoO_2_ with surface area of 115 m^2^ g^−1^ (1,308 and 1,594 mAh g^−1^, respectively). This result clearly indicates that the quality of the nanostructure of the mesoporous MoO_2_ material is very crucial to achieve a high Li-storage capacity that exceeds the theoretical value. Differences in the synthesis of mesoporous MoO_2_ materials with low and high surface areas are described in the Methods section. For more insight, scanning electron microscopic (SEM) images were obtained for the mesoporous MoO_2_ materials with different surface areas (Supplementary Fig. 6). Interestingly, there are co-existing large particles in the cases of the low surface area materials (39 and 76 m^2^ g^−1^; Supplementary Fig. 6c,e), whereas entire particles of the mesoporous MoO_2_ with the surface area of 115 m^2^ g^−1^ exhibit highly ordered mesostructures (Fig. 1a and Supplementary Fig. 6f). For comparison, the Li-storage behaviours of two different mixtures of bulk MoO_2_ and high-quality mesoporous MoO_2_ materials (resulting in *S*_BET_=21 m^2^ g^−1^ and 53 m^2^ g^−1^, respectively; Supplementary Figs 4, 5 and 6b,d and Supplementary Table 1) were also investigated, indicating that the mixtures give similar trends of Li-storage capacities depending on their surface areas.

**Figure 2:**
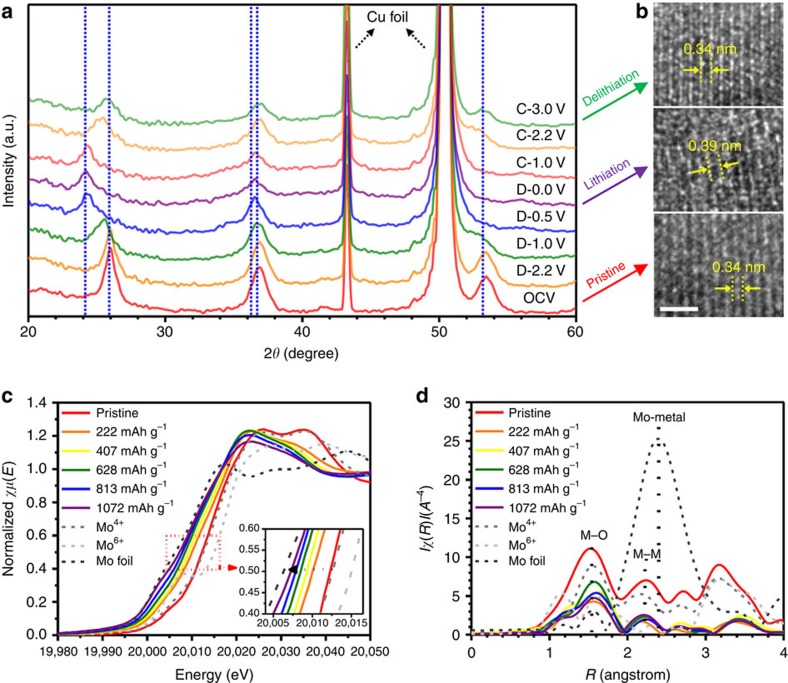
Structure evolution of Li storage in ordered mesoporous MoO_2_. (**a**) *Ex situ* XRD patterns of ordered mesoporous MoO_2_ during the first cycle as a function of depth of discharge and charge. (**b**) HRTEM images of mesoporous MoO_2_ before lithiation, after full lithiation and after full delithiation. Scale bar= 1.5 nm. (**c**) *In situ* XANES patterns and (**d**) *in situ* EXAFS patterns of ordered mesoporous MoO_2_ electrode for the first lithiation.

In order to gain insight on the physics and material science of Li storage in the present ordered mesoporous MoO_2_, we performed *ex situ* XRD analyses during the lithiation and delithiation of the ordered mesoporous MoO_2_. Figure 2a shows that Bragg peaks corresponding to MoO_2_ phase shifted to lower angles with an increase in the amount of Li stored, and returned to their initial scattering angles during the delithiation step, indicating highly reversible Li intercalation of the MoO_2_ host without collapse of its crystalline structure during cycling. High-resolution transmission electron microscopic (HRTEM) images (Fig. 2b) also show reversible changes of crystalline structure during the first cycle. We also observed reversible shifts of the Bragg peaks of MoO_2_ without the breakdown of the crystal structure even during the second cycle (Supplementary Fig. 7). It should be noted that we could not detect the evolution of any other phase related to the conversion reaction of MoO_2_, such as metallic Mo and Li_2_O, even after lithiation was electrochemically completed. These *ex situ* XRD analyses suggest that the present ordered mesoporous MoO_2_ does not follow the conventional conversion reaction mechanism, in which there is accommodation and release of Li ions by a Li intercalation mechanism. The XRD patterns obtained after the full lithiation (D−0.0 V in Fig. 2a, >7 mol of Li per 1 mol of MoO_2_) are very similar to the monoclinic Li_*x*_MoO_2_ phase, which means that the phase transition of crystal structures during the cycle is not the key for the unexpected high Li-storage capacity of the present mesoporous MoO_2_ material. In the HRTEM images (Supplementary Fig. 8), we found that the highly crystalline framework of the pristine mesoporous MoO_2_ material became a mixture of crystalline phase and amorphous phase upon the lithiation. This suggests that the amorphous phases formed between the crystalline domains may be critical for such high capacity of mesoporous MoO_2_. *In situ* Mo *K*-edge X-ray absorption near edge structure (XANES; Fig. 2c and Supplementary Fig. 9) spectra for the ordered mesoporous MoO_2_ electrode during lithiation show a clear shift of the absorption edge towards lower energy position, showing a change in the electronic structure of molybdenum from Mo^4+^ to a formal oxidation state close to Mo^1+^. This result indicates formation of another domain (probably the amorphous phase shown in the HRTEM image; Supplementary Fig. 8), in addition to the Li-intercalated monoclinic Li_*x*_MoO_2_ phase during the lithiation. Furthermore, *in situ* Mo *K*-edge extended X-ray absorption fine structure (EXAFS; Fig. 2d) spectra for the ordered mesoporous MoO_2_ show that the Mo–O and Mo–Mo peaks in the EXAFS spectra correspond to the coordination shells in MoO_2_ structure, excluding any possibility of metallic Mo phase. Besides the extraordinarily high capacity, these contradictory XANES and EXAFS results against the conventional conversion reaction of transition metal oxide anode materials strongly suggest that another reaction model for Li storage into the ordered mesoporous MoO_2_ should be considered in order to understand the origin of the extraordinary high Li-storage properties and the electrochemical reaction mechanism of the ordered mesoporous MoO_2_ with Li.

### Mechanism study on the Li-storage sites

**Figure 3:**
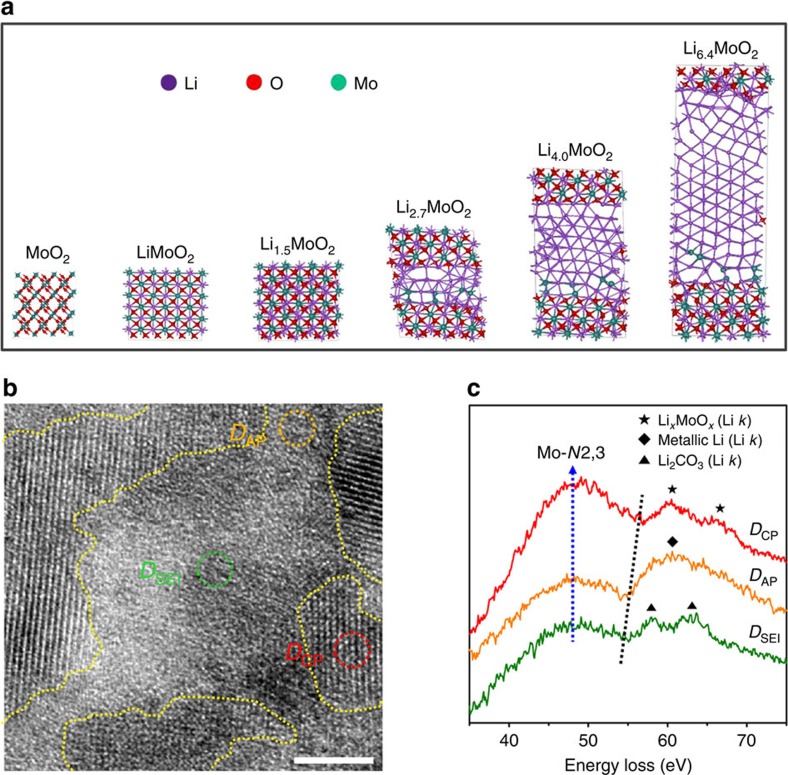
DFT calculations and STEM-EELS spectra. (**a**) Snapshots of the ordered mesoporous MoO_2_ electrode with the increase of Li inserted, calculated by DFT, and (**b**) top-view HRTEM image taken from a fully lithiated ordered mesoporous MoO_2_, demonstrating the formation of three different phases: crystalline phase (*D*_CP_), amorphous phase (*D*_AP_) and SEI (*D*_SEI_). Scale bar= 5 nm. (**c**) STEM-EELS spectra taken from areas *D*_CP_, *D*_AP_ and *D*_SEI_ of the fully lithiated ordered mesoporous MoO_2_. Dotted lines represent the peak position of Mo *N* edge (blue) and the onset of Li *K*-edge (black).

In order to obtain a theoretical perspective into the atomic movement and redox behaviour that occur upon lithiation of MoO_2_, we performed density functional theory (DFT) calculations on the Li-storage mechanism of the ordered mesoporous MoO_2_. Figure 3a shows that the lithiation process of MoO_2_ is almost the same for Li intercalation up to Li_1.5_MoO_2_ as evident from the small increase in unit cell volume (∼20%) and the retention of initial crystal structure, that is, the monoclinic MoO_2_ phase. However, further Li storage leads to phase separation of the lithiated MoO_2_ into a Li-rich phase (the amorphous domain in Supplementary Fig. 8) and Li-intercalated MoO_2_ crystalline phase (the crystalline domain in Supplementary Fig. 8). During the initial stage of this phase separation of lithiated MoO_2_, we found that additional Li atoms are deposited as nearest neighbours to a position occupied by a former Li atom. This near-neighbour Li atom siting is more energetically favourable as a new position for a Li atom than is a random position (Supplementary Fig. 10). As shown in Fig. 3a, the further lithiation results in volume increase during cycling without expansion in the lithiated crystalline Li_1.5_MoO_2_ domain. This total volume expansion by formation of Li-rich phase is probably an important characteristic that enables efficient Li storage in the ordered mesoporous MoO_2_. With an increase in the depth of the lithiation layer, only the Li-rich phase (formed between crystalline Li_*x*_MoO_2_ domains) reacts with Li ion, and the interatomic distance between Li atoms tends to decrease, suggesting that the electronic structure of the Li atoms turns into a metallic state. As seen in Supplementary Fig. 11, the calculated partial density of state of Li *s*-band clearly shows the change of the electronic states of Li into metallic states when *x*>1.5, which suggests that the metallic Li insertion between the crystalline Li_*x*_MoO_2_ domains causes the change in electronic structure.

It should be noted that this change in the electronic state of Li stored into a metal oxide differs from the interfacial Li-storage mechanism that has been used to explain the extra capacity of the transition metal oxide anode based on the conversion reaction mechanism^14^. According to the interfacial storage mechanism, extra Li ions are stored on the side of Li_2_O at the interface between Li_2_O and transition metal, which are formed through the fully conversion reaction of the transition metal oxide^14^. However, the Li-storage mechanism proposed in the present work for ordered mesoporous MoO_2_ does not follow the conversion reaction of MoO_2_ into Mo and Li_2_O, but rather retains the crystalline phases (MoO_2_ and Li_*x*_MoO_2_ during the cycle), with metallic Li accommodated between the Li-ion-intercalated MoO_2_ crystalline domains. This unexpected Li-storage mechanism for the extraordinarily high capacity of ordered mesoporous MoO_2_ can be attributed to the nanoscale pore-engineered structure, which offers a much higher surface area with a high metal oxide density. The thin MoO_2_ frameworks (∼7 nm) greatly shorten the diffusion length for the Li ions, thus enabling the electrochemical reaction that cannot occur in the bulk analogue.

In order to obtain additional direct experimental evidences for the two-phase reaction model proposed here (formation of both Li-ion-intercalated MoO_2_ crystalline phase and metallic Li-rich phase) along with the change of the electronic state of Li, we performed HRTEM and electron energy loss spectroscopy (EELS) studies on the fully lithiated and delithiated MoO_2_ electrode. The HRTEM image in Fig. 3b clearly shows that there are three different phases formed in the fully lithiated MoO_2_, that is, crystalline phase (*D*_CP_), amorphous phase (*D*_AP_) and solid electrolyte interface (SEI) phase (*D*_SEI_). The EELS data in scanning TEM (STEM) mode (STEM-EELS) taken at the crystalline area (*D*_CP_) show that the Li *K*-edge corresponds to Li_*x*_MoO_2_ (ref. 30), and this is closely relevant to the phase observed by *ex situ* XRD analyses, that is, Li-ion-intercalated MoO_2_ in Fig. 2a. However, STEM-EELS spectrum (Fig. 3c) taken at the amorphous area (*D*_AP_) shows that energy loss near edge structures of Li species are different from those taken at other areas (*D*_CP_ and *D*_SEI_) and corresponds to metallic Li^31^, implying that some of Li ions are accumulated as a form of metallic Li at the interface between nanosized crystalline Li_*x*_MoO_2_ domains. This Li storage as a metallic cluster resembles Li-storage mechanism of disordered carbon in the microspaces located at the edges of carbon clusters^32^,^33^,^34^,^35^.

In addition, as shown in Supplementary Fig. 12, the *Z*-contrast image and EELS spectra of O *K*-edge taken at the crystalline phase (*D*_CP_) and amorphous phase (*D*_AP_) strongly support this finding because the area of amorphous phase (*D*_AP_) shows darker contrast and lower oxygen intensity than the domain of the crystalline phase (*D*_CP_). Another STEM-EELS result on the area of *D*_SEI_ clearly shows a peak for Li_2_CO_3_ (Supplementary Fig. 13), which is one of the main components of SEI formed on the anode in the LIBs. As expected, the delithiated mesoporous MoO_2_ electrode does not contain any Li peaks, which suggests that it is reversibly returned to its initial state after the cycle (Supplementary Fig. 14). These EELS results strongly support the new reaction model of the ordered mesoporous MoO_2_ described in the present work.

### Cycle performance and rate capability

**Figure 4:**
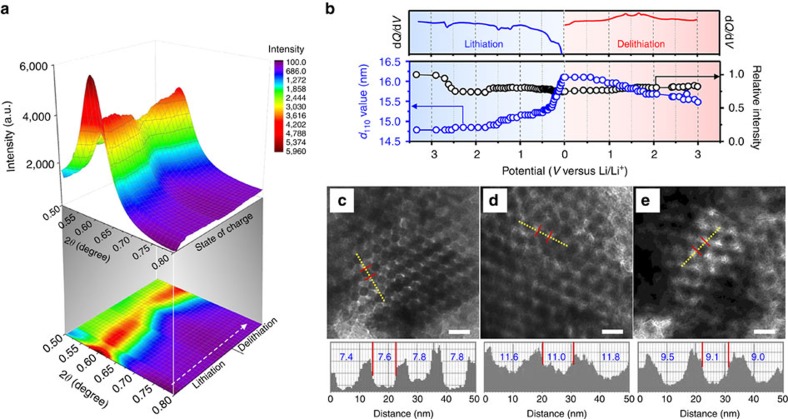
Pore structure of mesoporous MoO_2_ by *in operando* SAXS and *ex situ* TEM. (**a**) Color-coded 3D contour and projection map showing SAXS data collected from ordered mesoporous MoO_2_ during *in operando* experiment. (**b**) The changes in lattice parameter and resolved peak relative intensity calculated from the (110) reflection with the corresponding d*Q*/d*V* plot. Representative TEM images and framework thickness of ordered mesoporous MoO_2_ electrodes: (**c**) pristine, (**d**) lithiated and (**e**) delithiated. Scale bars= 20 nm.

Recently, we have developed an *in operando* small-angle X-ray scattering (SAXS) technique to investigate the nanostructural changes of ordered mesoporous electrode materials^36^. As shown in Fig. 4a,b and Supplementary Fig. 15, there is no significant changes in the *in operando* SAXS data of mesoporous MoO_2_ electrode during the lithiation and delithiation process (∼20% reduction of peak intensity and ∼25% increase of net volume change). These small changes in nanostructural properties indicate that the present mesoporous MoO_2_ electrode seems to follow the intercalation mechanism with additional Li-storage sites rather than the conventional conversion mechanism^36^. Figure 4c–e compares TEM images of the ordered mesoporous MoO_2_ structure during the first cycle. After complete lithiation, the diameter of the MoO_2_ frameworks increases from ∼7.8 to ∼11.8 nm. In addition to the diameter increase, there also should be a longitudinal volume expansion of MoO_2_ frameworks upon the lithiation (not experimentally observable). As shown by the *ex situ* XRD patterns and the DFT calculation on the lithiated MoO_2_ mesostructure (Figs 2a and 3a), the volume expansion can be attributed to the formation of both the Li-ion-intercalated crystalline phase and the amorphous metallic Li-rich phase during the lithiation. However, it is reasonable that the expansion associated with Li-ion intercalation is relatively smaller than that caused by the formation of the metallic Li-rich phase. This means that the volume expansion can be mainly attributed to the formation of the Li-rich phase, because the *d*_110_ spacing of crystalline MoO_2_ in the XRD patterns and the HRTEM images of Fig. 2a,b expanded by 14.7% from the pristine state during the transformation from MoO_2_ to Li_*x*_MoO_2_. It should be noted that, despite these volume changes during the first cycle, the ordered mesoporous MoO_2_ retained its mesostructure even after complete lithiation and returned to the initial state after the complete delithiation (Fig. 4c–e). It is also important to note that this high capacity of ordered mesoporous MoO_2_ electrode shows a significant improvement in capacity retention compared with the bulk material. As shown in Fig. 1h, the present mesoporous MoO_2_ electrode retains 121.9% of the initial capacity after 50 cycles (from 1,308 to 1,594 mAh g^−1^).

Cyclic voltammetry (CV) of the ordered mesoporous MoO_2_ electrode reveals the same tendency of highly stable electrochemical storage and removal of Li up to 30 cycles (Supplementary Fig. 16). Li-storage capacity of the ordered mesoporous MoO_2_ electrode shows the continuous increase with an increase of cycle numbers up to 20 cycles, and then reached the steady-state value (Supplementary Fig. 16b). Considering that this newly evolved couple of CV peaks could not be reported by other reports ever, the reduction (∼0.2 V) and oxidation peak (∼2.5 V), which can be also found at the differential capacity plots of the ordered mesoporous MoO_2_ electrode (Supplementary Fig. 17), would be associated with Li-storage reaction mechanism to account for high capacity of the ordered mesoporous MoO_2_ electrode presented here. It should be also noted that the rectangular CV profiles took shape with an increase of CV cycle numbers, indicating that Li-storage reaction in the ordered mesoporous MoO_2_ might resemble the electrochemical reaction observed in the supercapacitors rather than in the typical rechargeable batteries. Considering that under-potential deposition of metal ions is one of the typical super-capacitive electrochemical reactions, the rectangular shape of CV profiles observed in the mesoporous MoO_2_ electrode might be a circumstantial evidence to prove the storage of Li as a metallic phase in the ordered mesoporous MoO_2_ electrode.

Mo *K*-edge EXAFS spectra for MoO_2_ during electrochemical cycles are shown in Supplementary Fig. 18. We found that Mo–O peak from oxide phase is still major contribution in EXAFS spectra during cycles, even though the peak intensities are gradually reduced due to the cycle-induced structural disorder. It is notable that Mo–Mo peak from Mo metallic phase does not appear even after 10 cycles, clearly confirming no possibility to have the conversion reaction in MoO_2_ electrode.

Rate capability is also very important for electrode materials in addition to Li-storage capacity. We modified the mesostructure of the ordered mesoporous MoO_2_ by reducing the wall thickness from 7.9 to 7.0 nm and 5.5 to 4.0 nm (but for similar mesopore size of ∼18 nm, see Supplementary Figs 19–22 and Supplementary Tables 2 and 3), in order to examine the effects of framework thicknesses on the rate capability of mesoporous MoO_2_. As shown in Supplementary Fig. 23, the rate capability was slightly enhanced by reducing the framework thickness of the mesoporous MoO_2_ materials from 7.0 to 5.8 nm, which is probably by shortening the diffusion length for Li ion in the MoO_2_ framework. However, neither further reduction (5.1 nm) nor increase (7.8 nm) gives a positive effect on the rate capability. Research is underway to find the optimized mesopore size and framework thickness necessary to obtain the best electrochemical performance.

## Discussion

**Figure 5:**
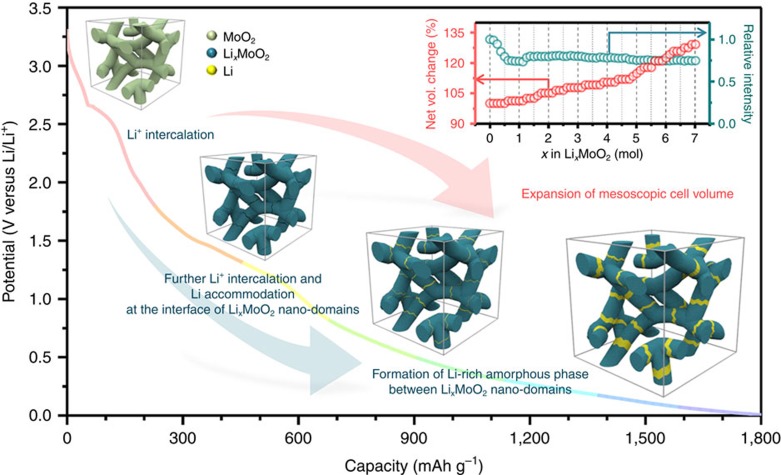
Schematic diagram of the reaction pathways and mesoscale morphology. Schematic diagram of the reaction pathways and the resulting products of ordered mesoporous MoO_2_ with respect to the amount of Li ion inserted.

We report a high-capacity Li-storage material, ordered mesoporous MoO_2_, with a capacity that exceeds the storage capacity predicted by the conventional Li-storage mechanism such as Li intercalation and/or conversion reaction of transition metal oxide. We suggest a Li-storage mechanism consisting of a Li-ion intercalation reaction and the formation of a metallic Li-rich phase between the Li-ion-intercalated MoO_2_ phase, based on first-principle calculations on the electronic structure of lithiated ordered mesoporous MoO_2_, along with DFT calculations, *in situ* X-ray absorption spectroscopies and HRTEM analyses combined with EELS studies of the reacted ordered mesoporous MoO_2_ (Fig. 5). In addition to the high capacity and excellent cycle performance of ordered mesoporous MoO_2_, the proposed Li-storage mechanism clearly shows how the nanoscale engineering impacts the associated physicochemical properties of the material for electrochemical mass storage applications. The present results enable a structural and electronic configuration that is, in a sense, a hybrid between the Li-intercalated compound anode and the metallic Li anode, with the inherent safety features of the former and a high power density approaching to that of the latter. The results further provide a more complete understanding of possible Li-storage mechanisms for transition metal oxides, and thus make possible the further advancement of ultrahigh capacity anode materials for Li rechargeable batteries.

## Methods

### Mesoporous silica template

In the present work, mesoporous silica, KIT-6, with cubic *Ia*3*d* mesostructure was used as the silica template for preparing mesoporous MoO_2_ materials. Pluronic triblock copolymer P123 (EO_20_PO_70_EO_20_, MW=5,800) was utilized as the structure-directing agent for synthesis of KIT-6. Typically, 90.0 g of P123 was dissolved in a mixture of 3,255 g of distilled water, 90 g of 1-BuOH (99.7 wt%, Aldrich) and 177 g of *c*-HCl (35 wt%, Aldrich). After stirring at 35 °C for 10 min, 193.5 g of tetraethylorthosilicate (98 wt%, Aldrich) was added to this solution under vigorous stirring. The resulting mixture was stirred for 24 h at 35 °C and subsequently kept in static condition at 100 °C for 24 h in an oven. The solid product was filtered, washed with double distilled water and dried at 100 °C overnight. The white powder, thus obtained, was washed with EtOH, dried at 80 °C for 12 h, and finally calcined under static air conditions at 550 °C for 3 h in order to remove the structure-directing agent. Mesopore size of the KIT-6 template calculated by Barrett–Joyner–Halenda method was 7.1 nm. KIT-6 materials with different pore sizes (5.1, 5.8 and 7.9 nm; see Supplementary Table 2 and Supplementary Fig. 19) were also synthesized using the above method, except for the hydrothermal treatment at 60, 80 and 140 °C, respectively.

### Highly ordered mesoporous MoO_2_ materials

The mesoporous MoO_2_ materials were synthesized by the nano-replication method using the KIT-6 as the mesoporous silica templates. An amount of 6.49 g of ammonium molybdate tetrahydrate ((NH_4_)_6_Mo_7_O_24_·4H_2_O, Aldrich) was dissolved in 8.4 g of distilled water. This precursor solution was infiltrated into 10.0 g of the calcined KIT-6 template by an incipient wetness method. After drying the composite at 80 °C for 24 h, the resulting material was heated to 500 °C under nitrogen atmosphere for 5 h for crystallization. After the heat treatment, the silica template was removed from the composite by a wet-etching process using a 20 wt% hydrofluoric acid (HF) solution. The resulting solid product was washed with distilled water and acetone several times, and then dried at 80 °C overnight in an oven. The mesoporous MoO_2_ materials, synthesized from the KIT-6 with different pore sizes, are denoted as meso-MoO_2_−*x* (where *x* means the synthesis temperature of KIT-6 templates). The mesoporous MoO_2_ materials with surface area of 39 and 76 m^2^ g^−1^ were prepared by following the same synthesis method with the above procedure, except for the precursor infiltration conditions. The precursor solutions were prepared by dissolving 20.7 g of (NH_4_)_6_Mo_7_O_24_·4H_2_O in 15 g of distilled water and 8.11 g of (NH_4_)_6_Mo_7_O_24_·4H_2_O in 10.5 g of distilled water for the mesoporous MoO_2_ with surface areas of 39 and 76 m^2^ g^−1^, respectively. Two mixed MoO_2_ electrodes was simply prepared by physically mixing the bulk MoO_2_ (Aldrich, *S*_BET_=0.23 m^2^ g^−1^) and the highly ordered mesoporous MoO_2_ (this work, *S*_BET_=115 m^2^ g^−1^). The weight ratios between the bulk and mesoporous MoO_2_ materials were 1:3 and 3:2 for the mixtures with surface areas of 21 m^2^ g^−1^ and 53 m^2^ g^−1^, respectively. The mixed MoO_2_ materials were thoroughly hand-mixed with mortar and pestle.

### Characterization

XRD patterns for the mesoporous silica and mesoporous MoO_2_ powder were obtained using a Rigaku D/MAX-2200 Ultima equipped with Cu Kα radiation at 30 kV and 40 mA. *Ex situ* XRD patterns of electrodes during the cycles were recorded in reflection mode using an X'Pert PRO equipped with Cu Kα radiation at 40 kV and 40 mA in the 20–60° (2*θ*) angular range. *In situ* XANES and EXAFS spectra were collected on beamline 10 C at Pohang Accelerator Laboratory. Energy calibration was carried out using the first inflection point of the spectrum of Mo metal foil as a reference (that is, Mo *K*-edge=20,000 eV). Reference spectra were simultaneously collected for each *in situ* spectrum using Mo metal foil. *In operando* SAXS experiments were carried out using BL 9 A U-SAXS beamline (Pohang Accelerator Laboratory) with a two-dimensional (2D) CCD detector (Rayonix SX165, USA) that was positioned 2 m away from the sample, which measured scattering in the 2*θ* range of 0.3–2.5 (*λ*=1.54 Å). The size of the focused beam was 300 μm in diameter and the energy of the beam was 11 keV. The 2D patterns of the mesoporous samples during electrochemical cycling were recorded with a 1-s exposure time and 8 s detector readout time. The 2D patterns were scanned with the FIT2D software package to obtain the 1D patterns in the form of the intensity versus 2*θ*. The storage ring was operated at 3.0 GeV with a ring current of 300 mA. SEM images were taken using a LEO Supra 55 field-emission SEM operating at an accelerating voltage of 15 kV. Ultra-high-resolution SEM images were obtained using a Hitachi UHR S 5500 FE-SEM operating at 30 kV. The main instruments we used in this study for microscopy were an aberration-corrected FEI TITAN field-emission TEM and a JEOL JEM 3010 TEM operated at an accelerating voltage of 300 kV. EELS analysis was performed in STEM mode using the field-emission TEM (TITAN) equipped with a high-resolution Gatan Tridieum 865ER imaging spectrometer. Because the MoO_2_ electrodes during lithiation and delithiation were sensitive to air and moisture, all the sampling process was carried out in an argon-filled glove box. For TEM observations, all the specimens were moved to the instruments using argon-filled plastic tubes and carefully transferred to the instruments within 10 s in order to minimize the air-contacting time. N_2_ adsorption–desorption isotherms were collected on a Micromeritics Tristar system at liquid N_2_ temperature. All of the samples were completely dried under vacuum at 100 °C for 24 h before the measurement. The specific BET (Brunauer–Emmett–Teller) surface areas were calculated from the adsorption branches in the range of relative pressure (*p*/*p*_0_)=0.05–0.20. The pore size distribution curves were obtained by the Barrett–Joyner–Halenda method on the basis of the adsorption branches. Total pore volumes were measured at *p*/*p*_0_=0.99.

### Electrochemistry

Electrodes were prepared by coating the slurries that contain the mesoporous MoO_2_ material as the active material powder, carbon black (Super-P, MMM) as the conducting agent and polyamideimide (Torlon 4,000 T, Solvay) as the binder in the weight ratio of 70:15:15 in *N*-methyl-2-pyrrolidone (Aldrich) solvent to produce an electrode slurry. The slurry was coated onto a Cu foil current collector using a Doctor-Blade technique. After the coating procedure, the electrodes were pressed and dried for 2 h at 200 °C under vacuum conditions. The electrodes thus obtained were cut into disks (12 mm in diameter). Coin-type cells (CR2016) were assembled in a dry room using Celgard 3,501 as the separator and Li foil as the counter and reference electrodes. On the basis of the current density of 0.1 C (=80 mAh g^−1^), all cells were tested at within a fixed voltage window (0.001–3.0 V) using a battery cycle tester (TOSCAT 4,000 series, Tokyo, Japan).

### Computational method

The first-principle calculations based on the DFT was performed using the VASP code. Initial structure of MoO_2_ is the 3 × 3 × 1 supercells including 36 Mo and 72 O atoms. The plane-wave cutoff energy was chosen to be 400 eV and appropriate *k*-points were chosen to ensure that the total energies are converged within a few meV. The exchange–correlation interactions between electrons were described by the generalized gradient approximation, and projector-augmented wave potentials were used for the description of ion–electron interactions. We used the conjugate gradient method for geometry optimizations, and the optimization procedure was truncated when the residual forces for the relaxed atoms were <0.03 eV per Å. In the calculation of Li_*x*_MoO_2_, Li atoms were inserted to the position of the energetically most stable site. During the lithiation, the atomic position and cell structures are fully relaxed simultaneously.

## Additional information

**How to cite this article:** Shon, J. K. *et al*. Discovery of abnormal lithium-storage sites in molybdenum dioxide electrodes. *Nat. Commun.* 7:11049 doi: 10.1038/ncomms11049 (2016).

## Supplementary Material

Supplementary Information Supplementary Figures 1-23 and Supplementary Tables 1-3.

## References

[b1] Nishi Y. The development of lithium ion secondary batteries. Chem. Rec. 1, 406–413 (2001). 11933247 10.1002/tcr.1024

[b2] Tarascon J.-M. & Armand M. Issues and challenges facing rechargeable lithium battery. Nature 414, 359–367 (2001). 11713543 10.1038/35104644

[b3] Ellis B. L., Lee K. T. & Nazar L. F. Positive electrode materials for Li-ion and Li-batteries. Chem. Mater. 22, 691–714 (2010).

[b4] Jeong G., Kim Y.-U., Kim H., Kim Y.-J. & Sohn H.-J. Prospective materials and applications for Li secondary batteries. Energ. Environ. Sci. 4, 1986–2002 (2011).

[b5] Lee K. T. & Cho J. Roles of nanosize in lithium reactive nanomaterials for lithium ion batteries. Nano Today 6, 28–41 (2011).

[b6] Huang J. Y. . *In situ* observation of the electrochemical lithiation of a single SnO_2_ nanowire electrode. Science 330, 1515–1520 (2010). 21148385 10.1126/science.1195628

[b7] Chan C. K. . High-performance lithium battery anodes using silicon nanowires. Nat. Nanotechnol. 3, 31–35 (2008). 18654447 10.1038/nnano.2007.411

[b8] Park C.-M., Kim J.-H., Kim H. & Sohn H.-J. Li-alloy based anode materials for Li secondary batteries. Chem. Soc. Rev. 39, 3115–3141 (2010). 20593097 10.1039/b919877f

[b9] Poizot P., Laruelle S., Grugeon S., Dupont L. & Tarascon J.-M. Nano-sized transition-metal oxides as negative-electrode materials for lithium-ion batteries. Nature 407, 496–499 (2000). 11028997 10.1038/35035045

[b10] Lee Y. J. . Fabricating genetically engineered high-power lithium-ion batteries using multiple virus genes. Science 324, 1051–1055 (2009). 19342549 10.1126/science.1171541

[b11] Armand M. & Tarascon J.-M. Building better batteries. Nature 451, 652–657 (2008). 18256660 10.1038/451652a

[b12] Grugeon S. . Particle size effects on the electrochemical performance of copoer oxides toward lithium. J. Electochem. Soc. 148, A285–A292 (2001).

[b13] Li H., Richter G. & Maier J. Reversible formation and decomposition of LiF clusters using transition metal fluorides an precursors and their application in rechargeable Li batteries. Adv. Mater. 15, 736–739 (2003).

[b14] Jamnik J. & Maier J. Nanocrystallinity effects in lithium battery materials. Phys. Chem. Chem. Phys. 5, 5215–5220 (2003).

[b15] Hu Y.-Y. . Origin of additional capacities in metal oxide lithium-ion battery electrodes. Nat. Mater. 12, 1130 (2013). 24185759 10.1038/nmat3784

[b16] Cheng F., Liang J., Tao Z. & Chen J. Functional materials for rechargeable batteries. Adv. Mater. 23, 1695–1715 (2011). 21394791 10.1002/adma.201003587

[b17] Bruce P. G., Scrosati B. & Tarascon J.-M. Nanomaterials for rechargeable lithium batteries. Angew. Chem. Int. Ed. 47, 2930–2946 (2008). 10.1002/anie.200702505 18338357

[b18] Liu C., Li F., Ma L.-P. & Cheng H.-M. Advanced materials for energy storage. Adv. Mater. 22, E28–E62 (2010). 20217798 10.1002/adma.200903328

[b19] Kresge C. T., Leonowicz M. E., Roth W. J., Vartuli J. C. & Beck J. S. Ordered mesoporous molecular sieves synthesized by a liquid-crystal template mechanism. Nature 359, 710–712 (1992).

[b20] Zhao D. . Triblock copolymer syntheses of mesoporous silica with periodic 50 to 300 angstrom pores. Science 279, 548–552 (1998). 9438845 10.1126/science.279.5350.548

[b21] Joo S. H. . Ordered nanoporous arrays of carbon supporting high dispersions of platinum nanoparticles. Nature 412, 169–172 (2001). 11449269 10.1038/35084046

[b22] Lee H. I. . Spontaneous phase separation mediated synthesis of 3D mesoporous carbon with controllable cage and window size. Adv. Mater. 23, 2357–2361 (2011). 21495082 10.1002/adma.201003599

[b23] Chang H., Joo S. H. & Pak C. Synthesis and characterization of mesoporous carbon for fuel cell applications. J. Mater. Chem. 17, 3078–3088 (2007).

[b24] Yue W. . Syntheses, Li insertion and photoactivity of mesoporous crystalline TiO_2_. Adv. Func. Mater. 19, 2826–2833 (2009).

[b25] Ren Y., Jardwick L. J. & Bruce P. G. Lithium intercalation into mesoporous anatase with an ordered 3D pore structure. Adv. Mater. 49, 2570–2574 (2010). 10.1002/anie.200907099 20209547

[b26] Ren Y., Armstrong A. R., Jiao F. & Bruce P. G. Influence of size on the rate of mesoporous electrodes for lithium batteries. J. Am. Chem. Soc. 132, 996–1004 (2010). 20039669 10.1021/ja905488x

[b27] Shi Y. . Ordered mesoporous metallic MoO_2_ materials with highly reversible lithium storage capacity. Nano Lett. 9, 4215–4220 (2009). 19775084 10.1021/nl902423a

[b28] Sohn J. K. . Nano-propping effect of residual silicas on reversible lithium storage over highly ordered mesoporous SnO_2_ materials. J. Mater. Chem. 19, 6727–6732 (2009).

[b29] Chen C., Ding N., Wang L., Yu Y. & Lieberwirth I. Some new facts on electrochemical reaction mechanism for transition metal oxide electrodes. J. Power Sources 189, 552–556 (2009).

[b30] Cosandey F. in Microscopy: Science, Technology, Applications and Education eds Méndez-Vilas A., Díaz J.) Vol. 3, 1662–1666Formatex (2010).

[b31] Hightower A., Ahn C. C., Fultz B. & Rez P. Electron energy-loss spectrometry on lithiated graphite. Appl. Phys. Lett. 77, 238–240 (2000).

[b32] Mochida I., Ku C.-H. & Korai Y. Anodic performance and insertion mechanism of hard carbons prepared from synthetic isotropic pitches. Carbon N. Y. 39, 399–410 (2001).

[b33] Dahn J. R., Zheng T., Liu Y. & Xue J. S. Mechanisms for lithium insertion in carbonaceous materials. Science 270, 590–593 (1995).

[b34] Mabuchi A., Tokumitsu K., Fujimoto H. & Kasuh T. Charge-discharge characteristics of the mesocarbon miocrobeads heat-treated at different temperatures. J. Electrochem. Soc. 142, 1041–1046 (1995).

[b35] Mochida I., Ku C.-H., Yoon S. H. & Korai Y. Anodic performance and mechanism of mesophase-pitch-derived carbons in lithium ion batteries. J. Power Sources 75, 214–222 (1998).

[b36] Park G. O. . *In operando* monitoring of the pore dynamics in ordered mesoporous electrode materials by small angle X-ray scattering. ACS Nano 9, 5470–5477 (2015). 25869353 10.1021/acsnano.5b01378

