# Supplementary material for: Discovery of abnormal lithium-storage sites in molybdenum dioxide electrodes
Source: Nat Commun. 2016 Mar 22;7:11049. doi: 10.1038/ncomms11049 (PMC4804172; doi:10.1038/ncomms11049)
Supplement: Supplementary Information — Supplementary Figures 1-23 and Supplementary Tables 1-3. [file ncomms11049-s1.pdf]

Supplementary Figures

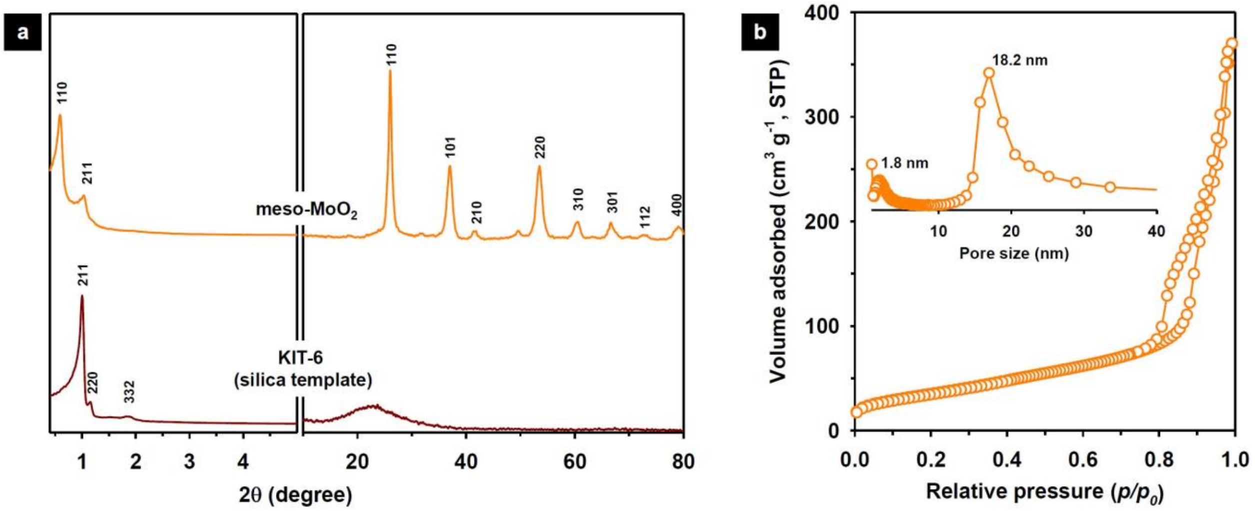

Supplementary Figure 1. (**a**) XRD patterns of mesoporous silica template, KIT-6, and mesoporous MoO_2 obtained by nano-replication method. The KIT-6 template exhibits typical XRD peaks that are characteristic of a 3-D cubic (*Ia*3*d*) mesostructure. In the case of mesoporous MoO_2, however, a new appeared at the low angle region, which corresponds to the position of the 110 reflection for *Ia*3*d* symmetry. Since the *Ia*3*d* symmetry are not allowed to have the 110 reflection, the presence of the new XRD peak indicates that the cubic *Ia*3*d* mesostructure is transformed to the tetragonal $I4_1/a$ (or lower) mesostructures or a single gyroid structure after the removal of silica template. The wide-angle XRD pattern of mesoporous MoO_2 shows several peaks that are characteristic of pseudotetragonal rutile MoO_2 phase (JCPDS: 02-0422). The average size of a MoO_2 domain, calculated from XRD line-broadening by Scherrer formular, is about 7 nm, which is very similar to the pore size of KIT-6 template as well as the wall thickness of mesoporous MoO_2 measured from TEM images. (**b**) N_2 adsorption-desorption isotherms for the mesoporous MoO_2 and the corresponding BJH pore size distribution curve. The N_2 sorption isotherm is a typical type-IV isotherm with hysteresis, which is characteristic of mesoporous materials. The BET surface area is 115 m^2g^{-1}, and a well-defined step appears in the adsorption-desorption curves around a relative pressure, p/p_0, of 0.8 – 0.9. The BJH pore size obtained from the adsorption branch is about 18.2 nm, which is much larger than the wall thickness of the silica template. This is also evidence for the phase transformation from *Ia*3*d* to the others after silica removal, as expected from the XRD patterns. The BJH pore size distribution curve also shows small amount of mesopore with about 2 nm in diameter, which probably arise from the silica framework of KIT-6 template.

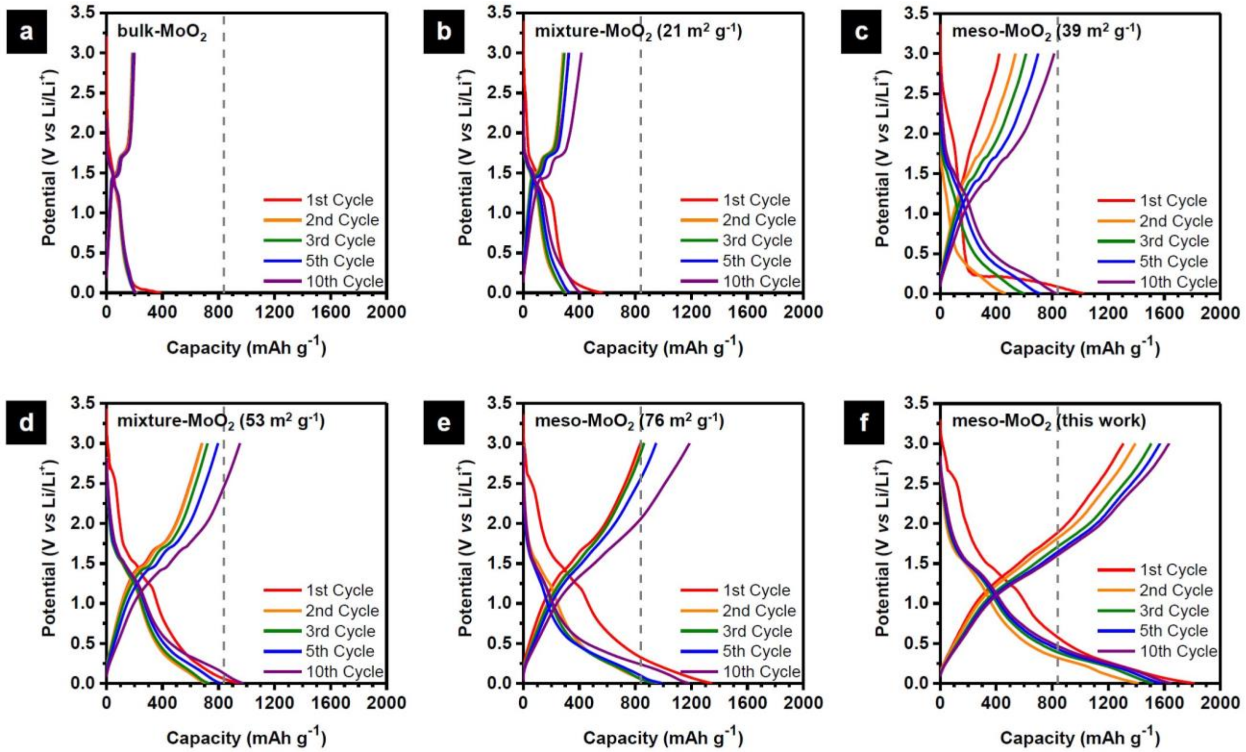

Supplementary Figure 2. Voltage profiles of various MoO_2 electrode materials: (**a**) bulk MoO_2, (**b**) physical mixture of MoO_2 (S_{BET} = 21 m^2 g^{-1}), (**c**) mesoporous MoO_2 (S_{BET} = 39 m^2 g^{-1}), (**d**) physical mixture of MoO_2 (S_{BET} = 53 m^2 g^{-1}), (**e**) mesoporous MoO_2 (S_{BET} = 76 m^2 g^{-1}) and (**f**) mesoporous MoO_2 (this work, S_{BET} = 115 m^2 g^{-1}).

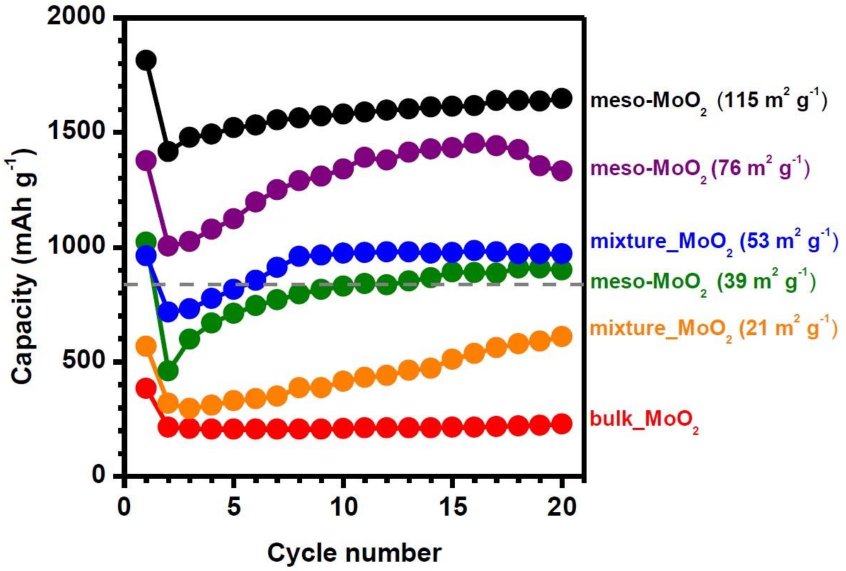

Supplementary Figure 3. Cyclic performaces of various MoO_2 electrode materials.

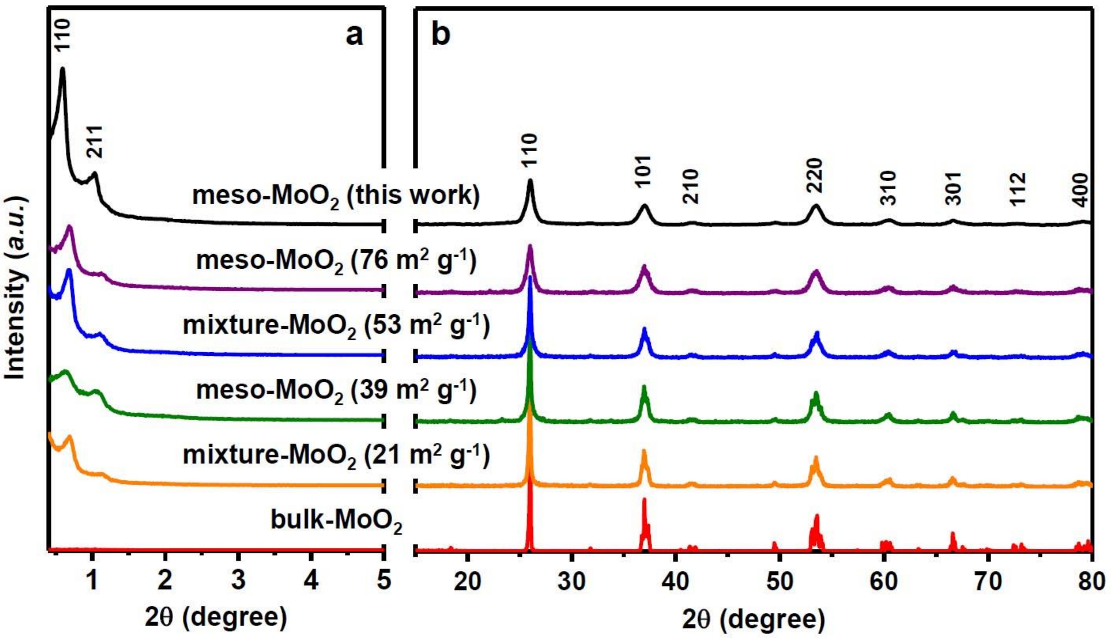

Supplementary Figure 4. Small- and wide-angle XRD patterns of various MoO_2 electrode materials.

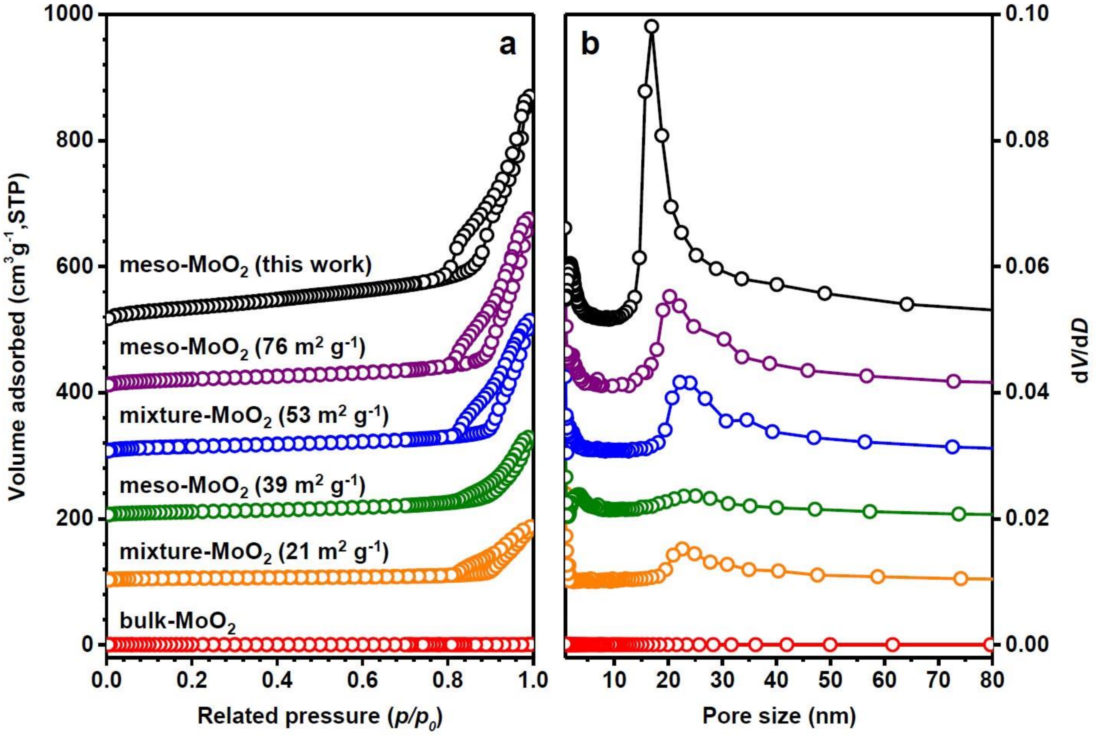

Supplementary Figure 5. (a) N_2 adsorption-desorption isotherms of various MoO_2 electrode materials, and (b) the corresponding BJH pore size distribution curves.

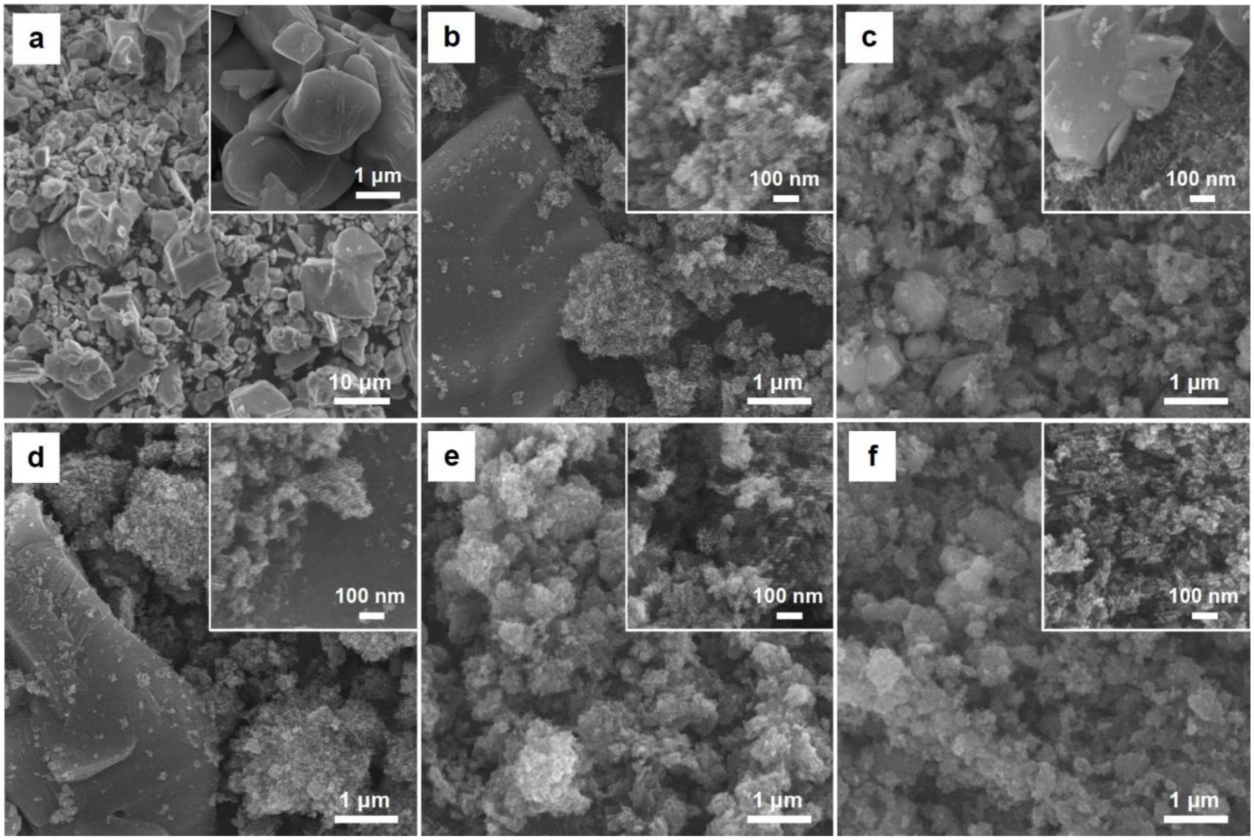

Supplementary Figure 6. SEM images of various MoO_2 electrode materials: (**a**) bulk MoO_2, (**b**) physical mixture of MoO_2 (S_{BET} = 21 m^2 g^{-1}), (**c**) mesoporous MoO_2 (S_{BET} = 39 m^2 g^{-1}), (**d**) physical mixture of MoO_2 (S_{BET} = 53 m^2 g^{-1}), (**e**) mesoporous MoO_2 (S_{BET} = 76 m^2 g^{-1}) and (**f**) mesoporous MoO_2 (this work, S_{BET} = 115 m^2 g^{-1}).

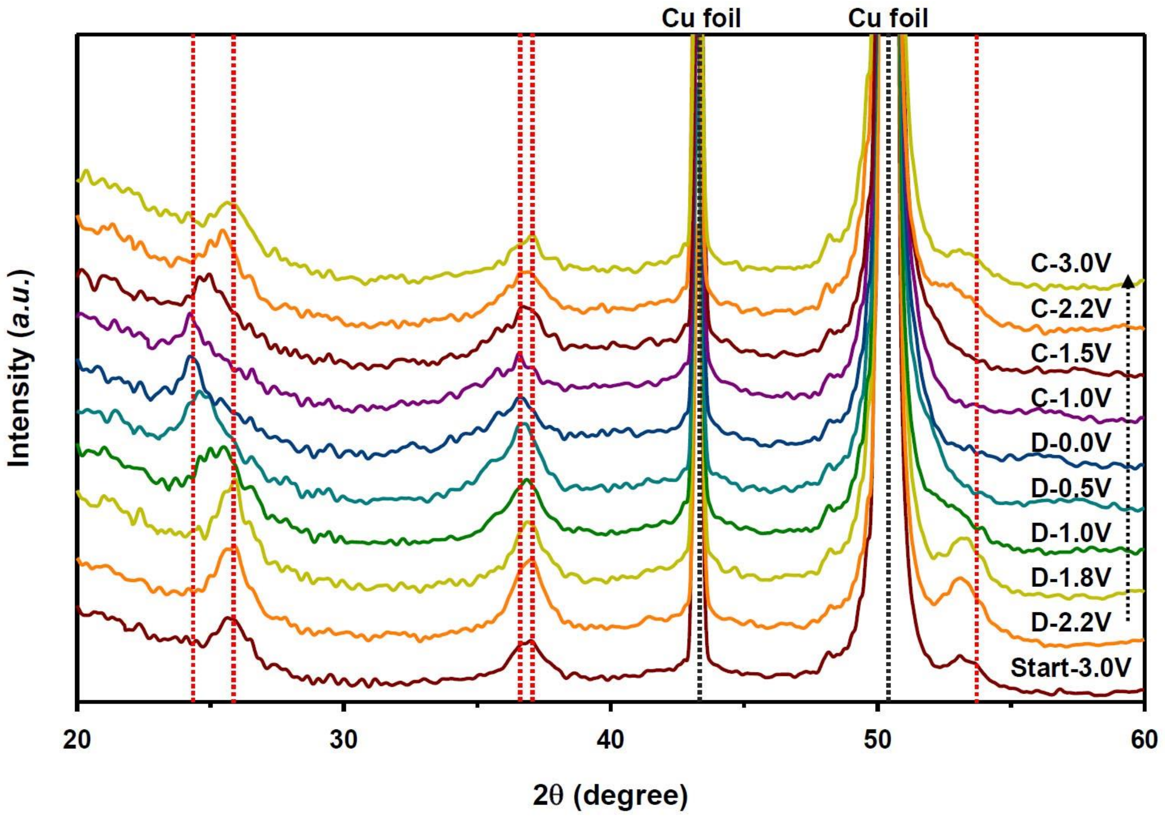

Supplementary Figure 7. *Ex situ* XRD patterns of mesoporous MoO_2 during the second cycle.

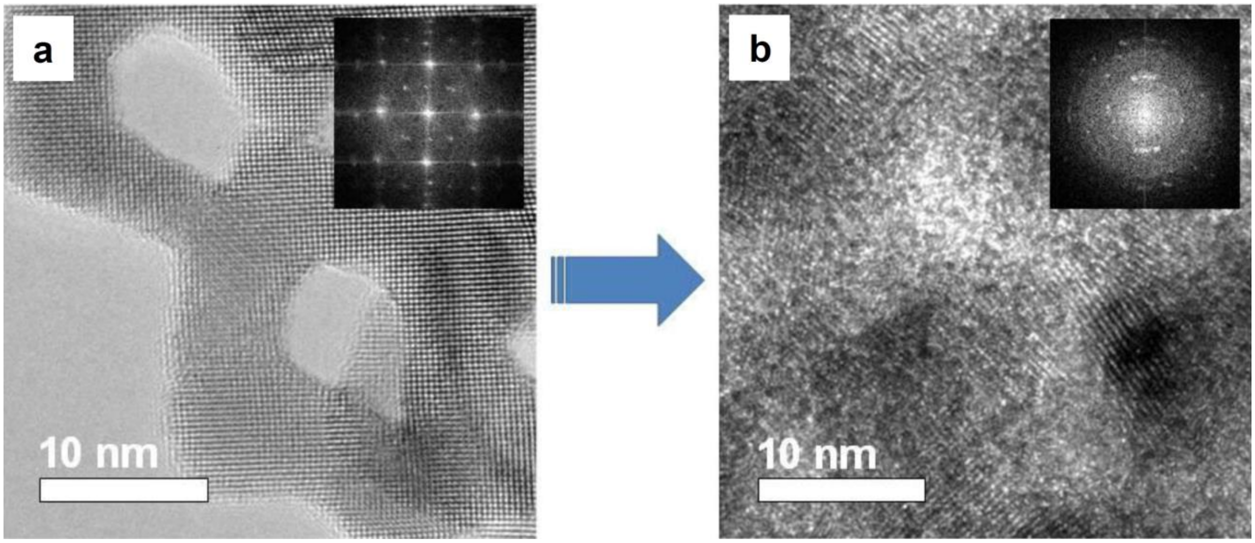

Supplementary Figure 8. HRTEM images of mesoporous MoO_2 (**a**) before lithiation and (**b**) after lithiation.

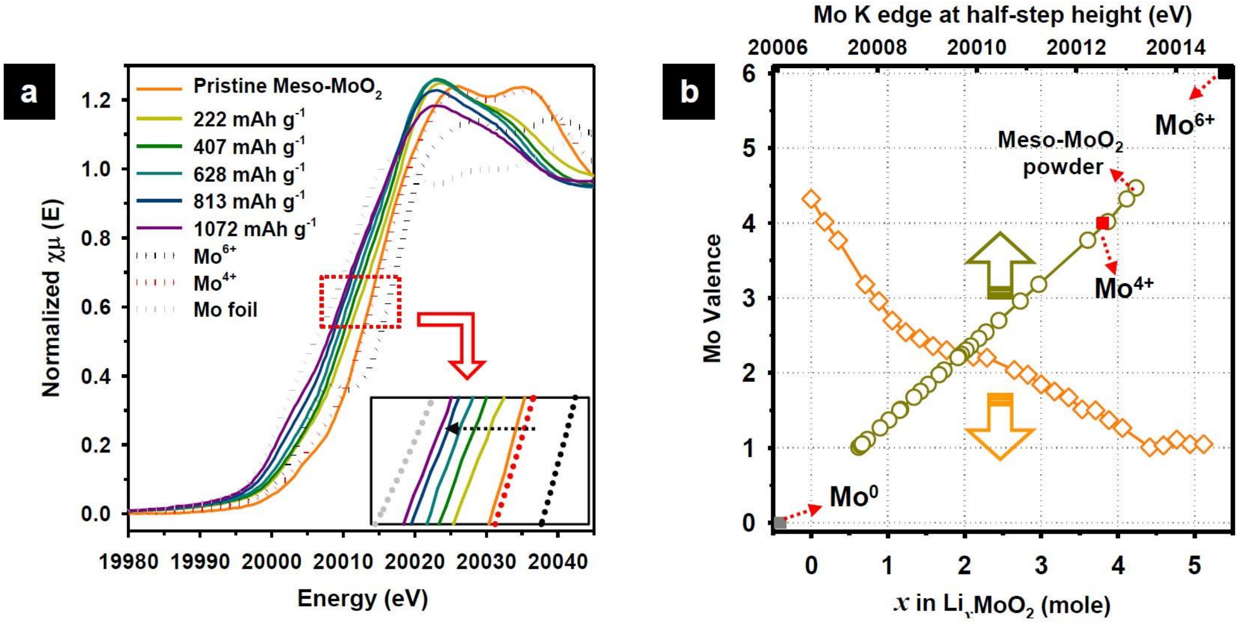

Supplementary Figure 9. (**a**) *in situ* XANES spectra obtained from Mo *K*-edge, and (**b**) relationship of average Mo valence vs. Mo *K*-edge and mole number of Li (x) in Li_xMoO_2 with the increase of depth of the lithiation. Average valences of Mo species (y-axis in (**b**)) were calculated by using *K*-edge value of reference materials of bulk MoO_3, MoO_2 and metallic Mo at half-step height.

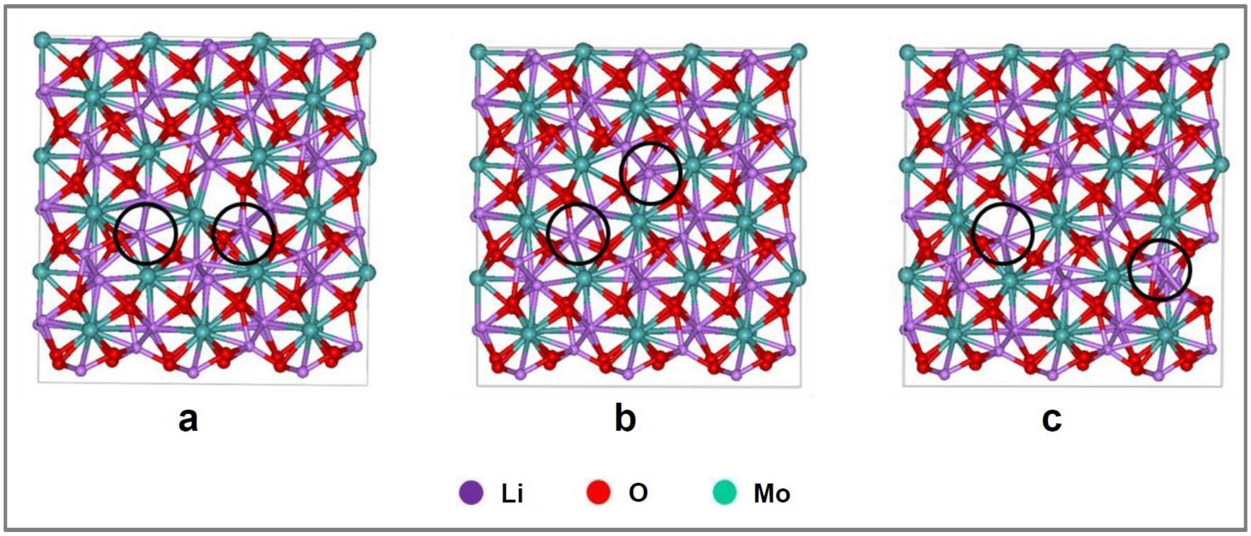

Supplementary Figure 10. Initial stage of Li intercalated position at $Li_{1.5+x}MoO_2$. (**a**) two Li intercalated bridge over Mo atom (**b**) two Li intercalated bridge over 0 atom (**c**) two Li intercalated separated position in this case. The case (**a**) is the most favorable position of Li intercalation.

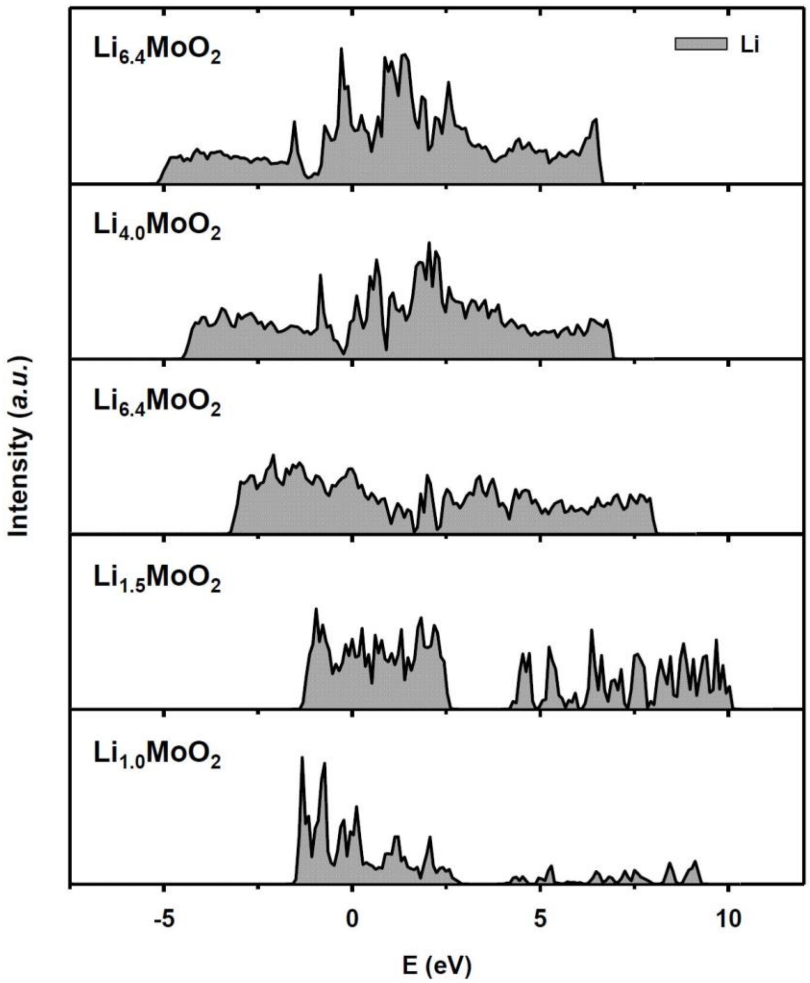

Supplementary Figure 11. Partial density of states (PDOS) of Li s band by DFT calculation.

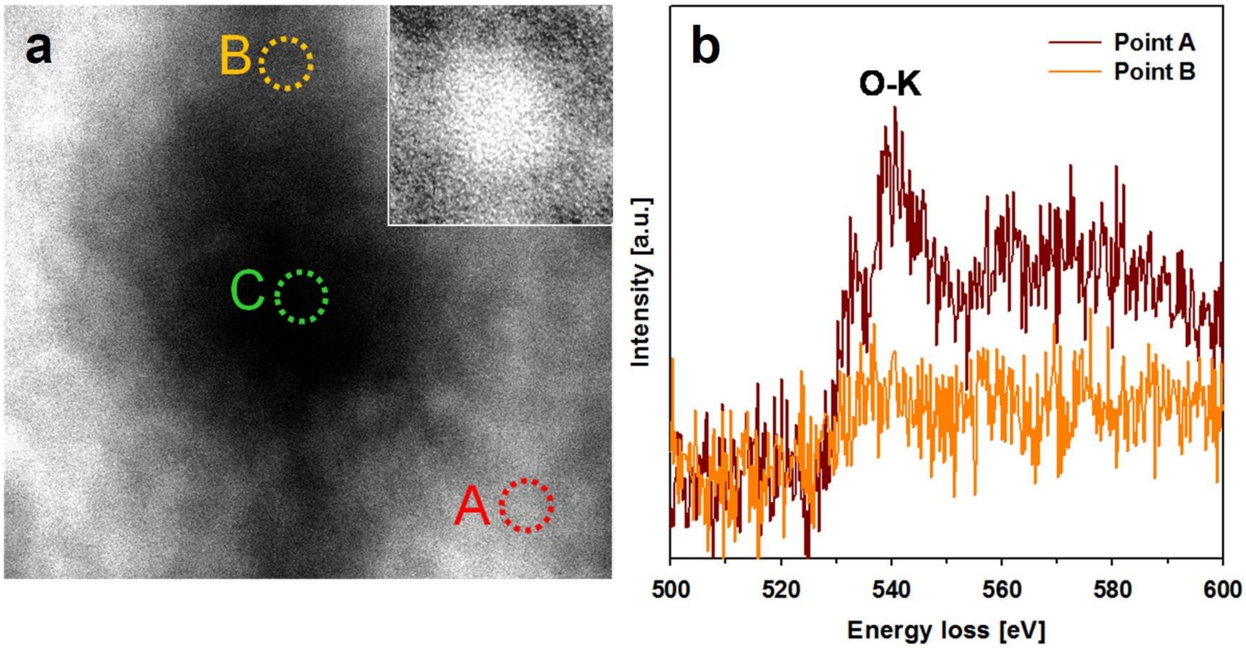

Supplementary Figure 12. Magnified Z-contrast image of (**a**) the fully lithiated mesoporous MoO_2 and (**b**) its core-excitation EELS spectra of O K-edge taken at areas A and B. The inset in (**a**) shows a TEM image taken from the same area.

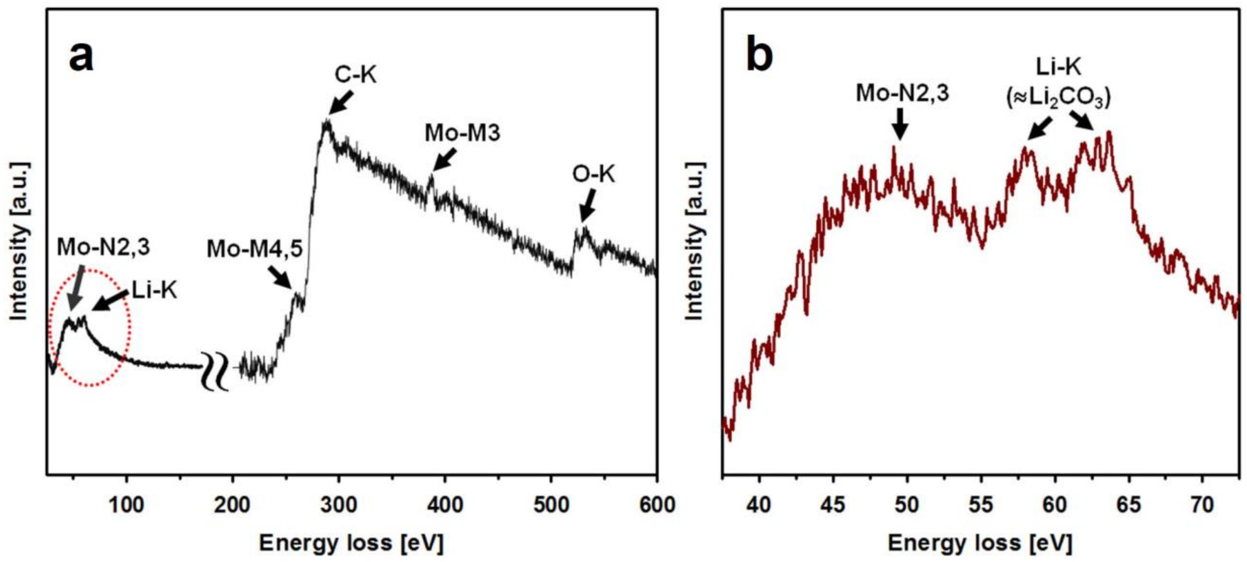

Supplementary Figure 13. (a) EELS spectrum obtained at the area C shown in Fig. S9, and (**b**) magnified EELS spectrum of (**a**) showing details on Mo-*N* edge and Li-*K* edge.

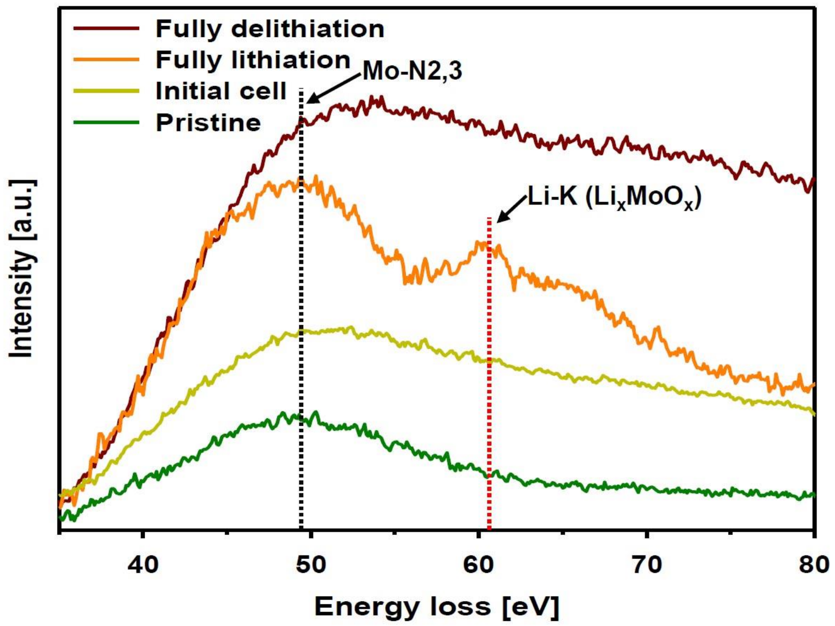

Supplementary Figure 14. Changes of EELS spectra obtained from the crystalline MoO_2 areas before and after lithiation and delithiation processes.

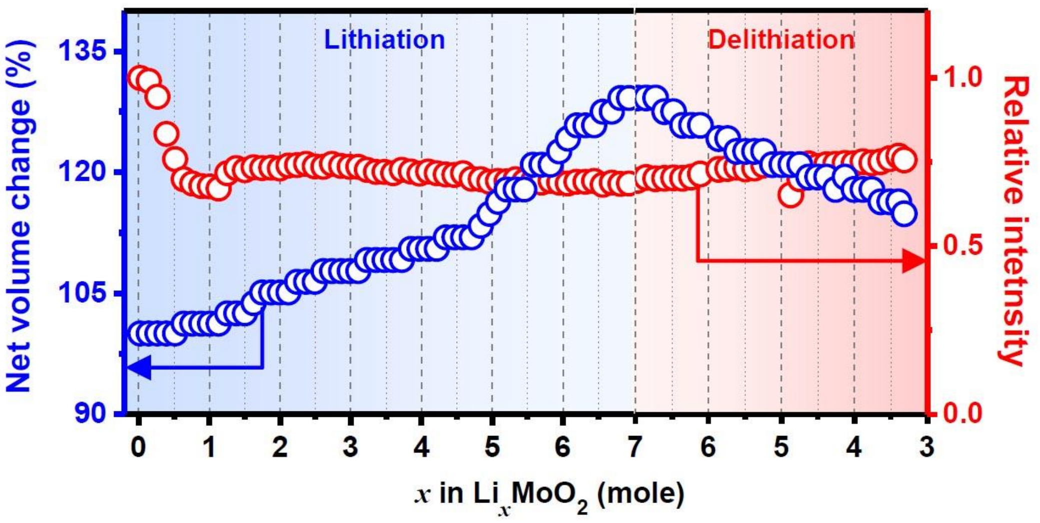

Supplementary Figure 15. Net volume change and resolved peak relative intensity with contained lithium in the mesoporous MoO_2 electrode during lithiation-delithiation process.

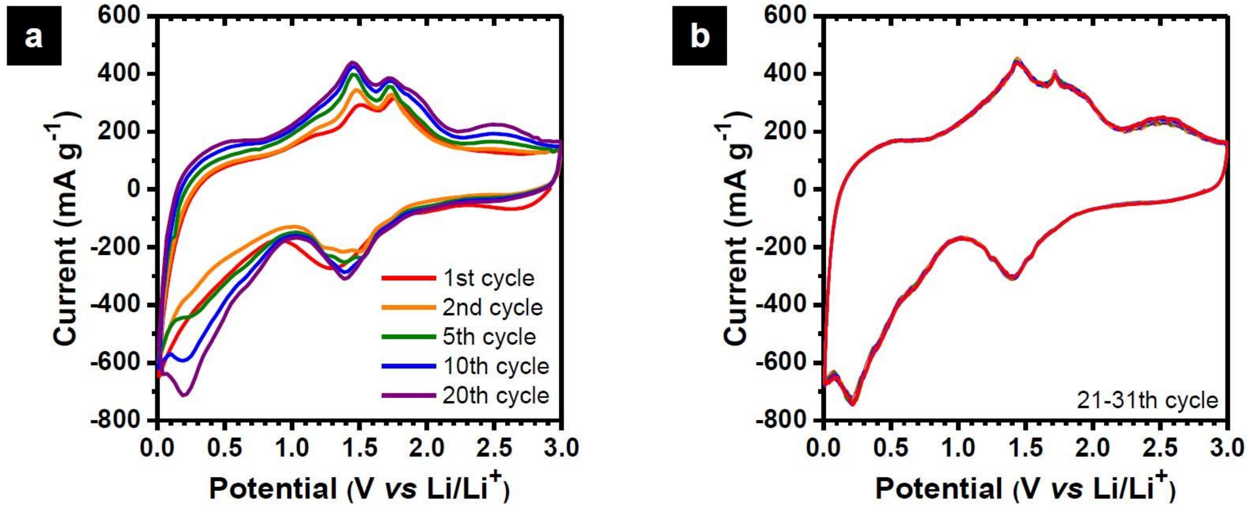

Supplementary Figure 16. Cyclic voltammetry profiles of the mesoporous MoO_2 electrode **(a)** from 1st to 20th cycle, and **(b)** from 21st to 30th cycle.

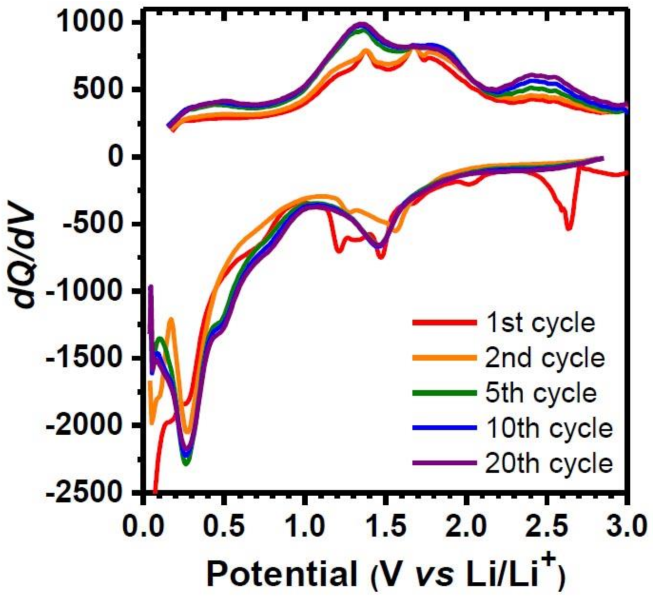

Supplementary Figure 17. dQ/dV data of mesoporous MoO_2 electrode from 1st to 20th cycle.

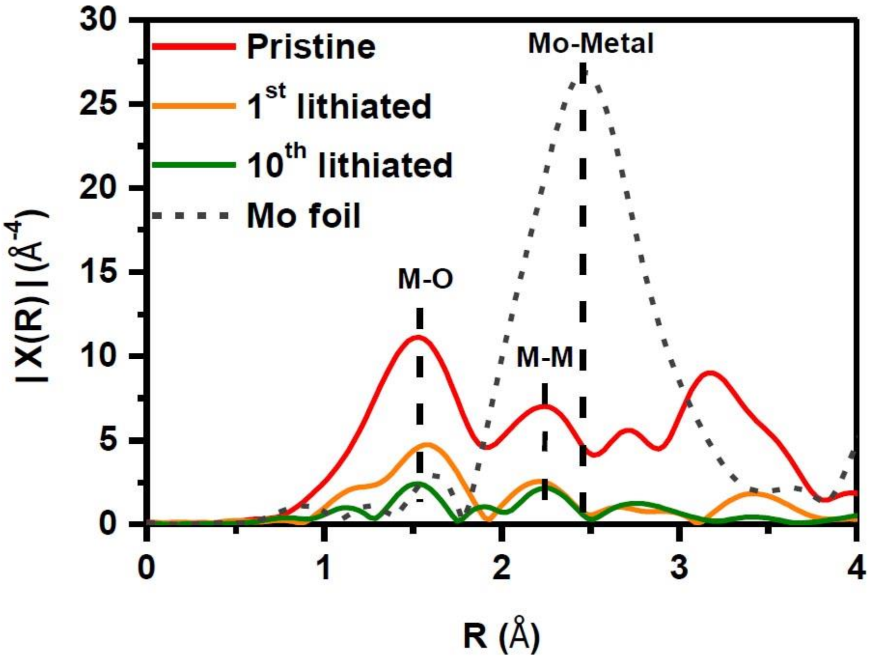

Supplementary Figure 18. Mo *K*-edge EXAFS data of the mesoporous MoO_2 electrode.

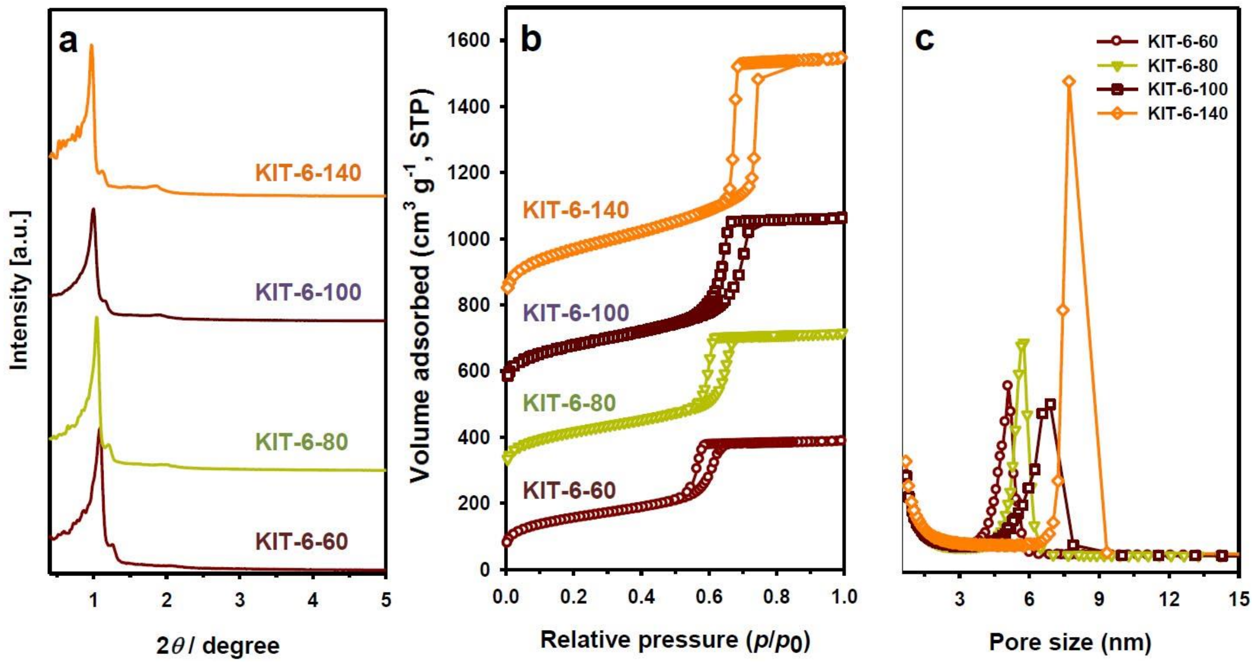

Supplementary Figure 19. (**a**) XRD patterns, (**b**) N_2 sorption isotherms and (**c**) the corresponding pore size distribution curves of KIT-6 templates synthesized at different hydrothermal temperatures.

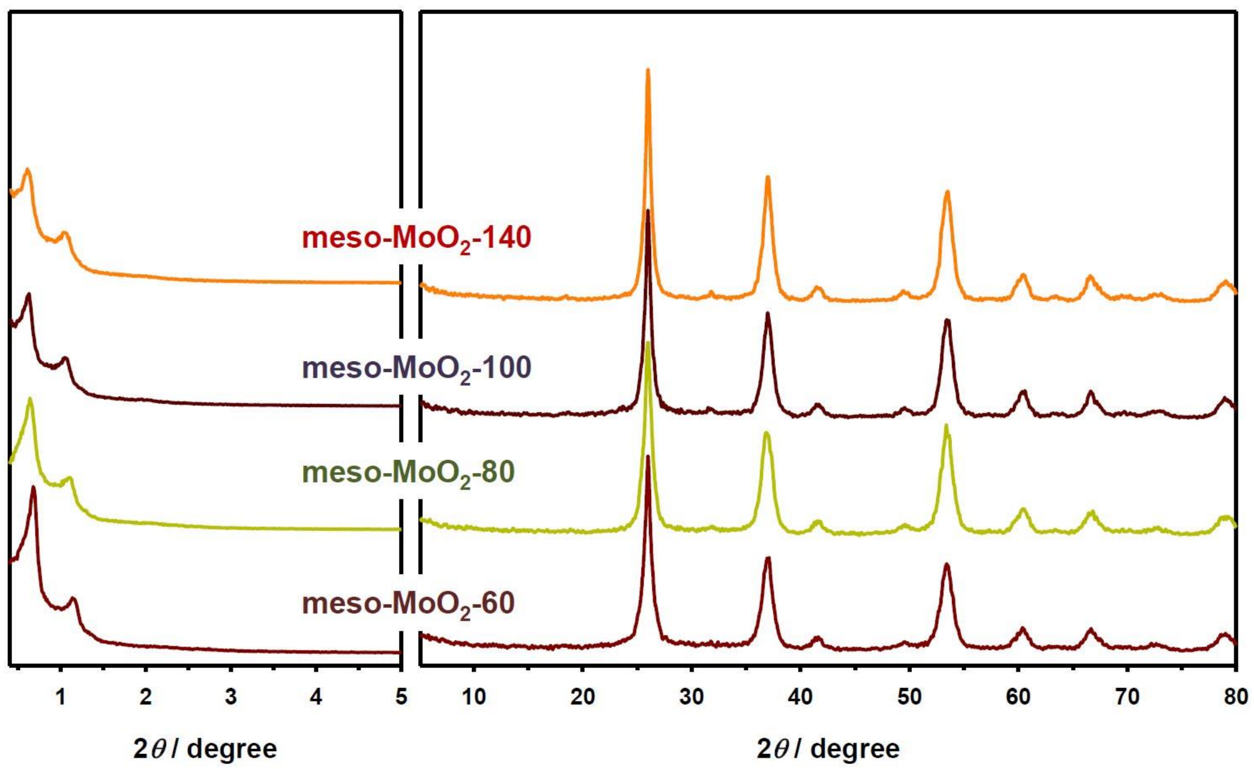

Supplementary Figure 20. XRD patterns of mesoporous MoO_2 materials with different framework thicknesses.

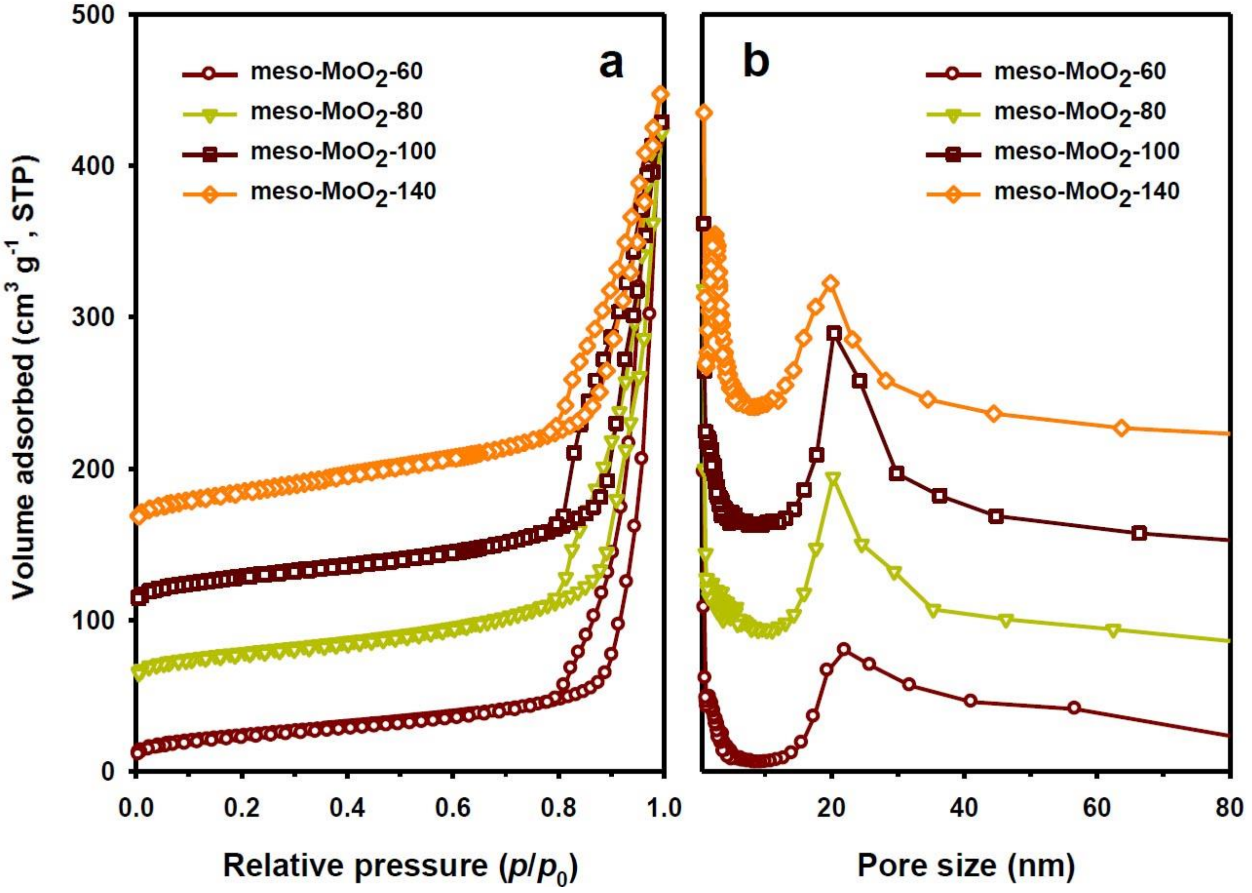

Supplementary Figure 21. (**a**) N_2 adsorption-desorption isotherm and (**b**) the corresponding BJH pore size distribution curve for mesoporous MoO_2 with the controlled framework thickness.

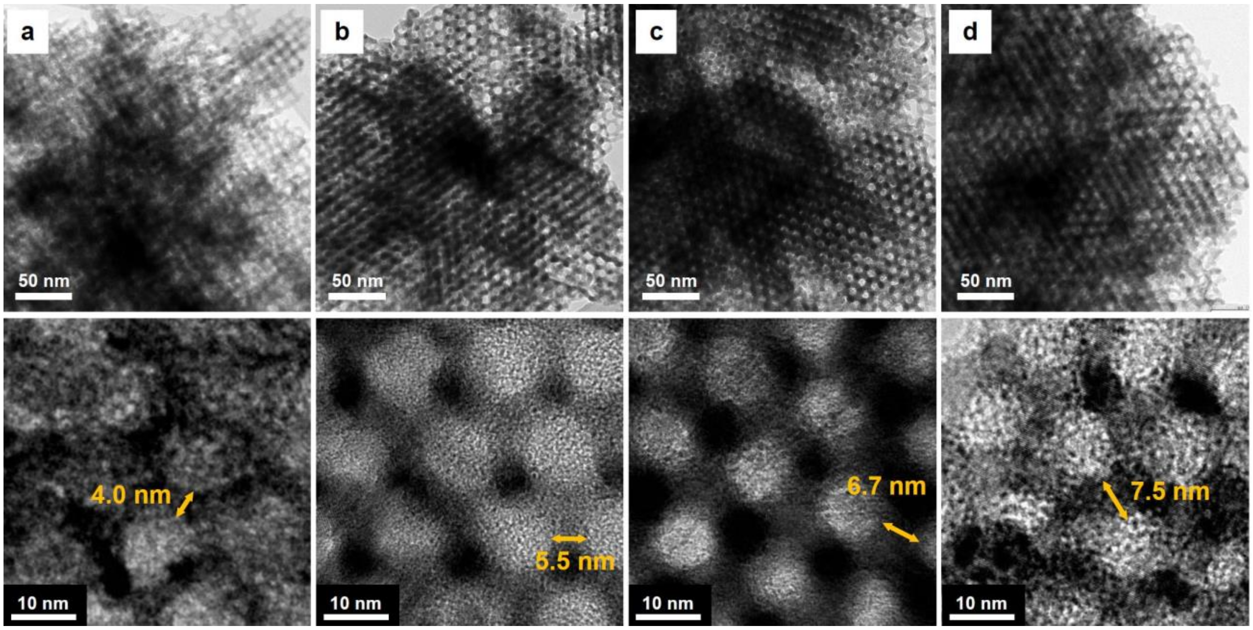

Supplementary Figure 22. TEM images of mesoporous MoO_2 materials with different framework thicknesses.

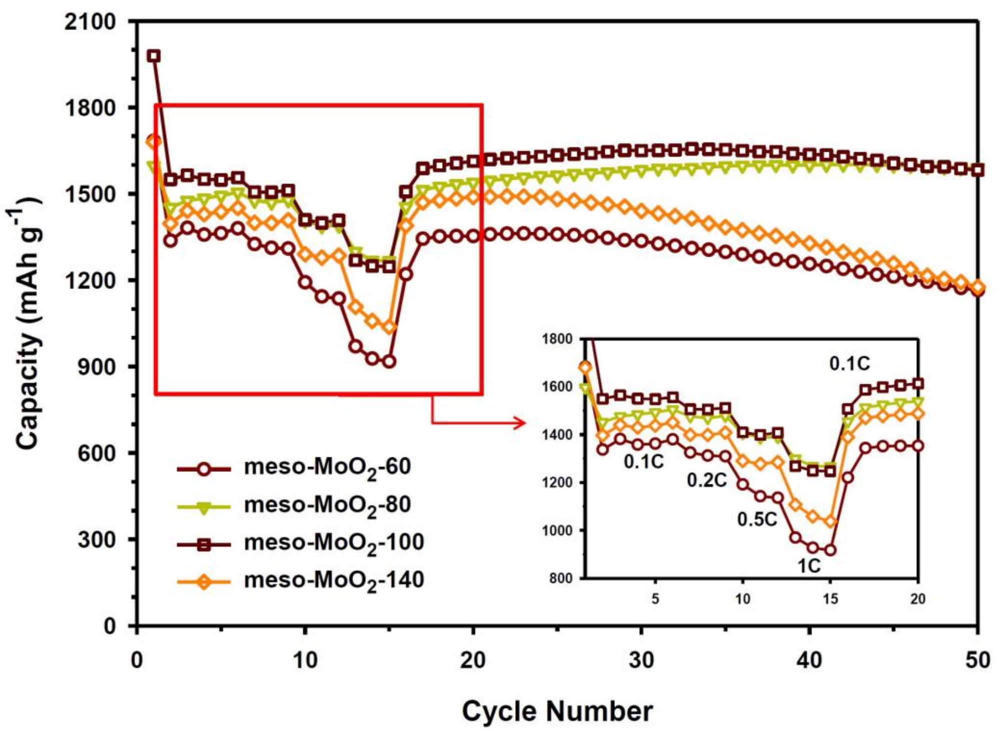

Supplementary Figure 23. Cycle performances of mesoporous MoO_2 with different framework thickness for rate capability at current rate from 0.1 C to 1 C in 1.3 M $LiPF_6$ (EC/DEC = 3/7, by volume ratio).

Supplementary Tables

Supplementary Table 1. Physical properties of MoO_2 with the controlled surface area.

Materials	$S_{BET}{}^{a}$ (m^2 g^{-1})	$V_{tot}{}^{b}$ (cm^3 g^{-1})
bulk-MoO_2	0.23	0.001
mixture-MoO_2	21	0.13
meso-MoO_2	39	0.20
mixture-MoO_2	53	0.33
meso-MoO_2	76	0.43
meso-MoO_2 (this work)	115	0.57

[a] BET surface areas calculated in the range of relative pressure (p/p_0) = 0.05–0.20
[b] Total pore volume measured at p/p_0 = 0.99

Supplementary Table 2. Physical properties of KIT-6 template synthesized at different temperatures.

Materials	a^a (nm)	$S_{BET}{}^b$ (m^2 g^{-1})	$V_{tot}{}^c$ (cm^3 g^{-1})	$D_{pore}{}^d$ (nm)
KIT-6-60	19.84	477	0.51	5.1
KIT-6-80	20.79	609	0.72	5.8
KIT-6-100	21.62	738	1.00	7.1
KIT-6 -140	22.29	865	1.33	7.9

[a] Lattice parameters calculated from XRD peak for the materials.
[b] BET surface areas calculated in the range of relative pressure (p/p_0) = 0.05–0.20
[c] Total pore volume measured at p/p_0 = 0.99
[d] BJH pore size calculated from the adsorption branches

Supplementary Table 3. Physical properties of mesoporous MoO_2 with the controlled framework thickness.

Materials	S_{BET}[a] (m^2 g^{-1})	V_{tot}[b] (cm^3 g^{-1})	$T_{framework}$[c] (nm)
meso-MoO_2-60	83	0.64	5.1
meso-MoO_2-80	102	0.58	5.8
meso-MoO_2-100	115	0.51	7.0
meso-MoO_2-140	126	0.46	7.5

[a] BET surface areas calculated in the range of relative pressure (p/p_0) = 0.05–0.20
[b] Total pore volume measured at p/p_0 = 0.99
[c] Framework thickness determined from TEM images
